# Genetic risk of major depressive disorder: the moderating and mediating effects of neuroticism and psychological resilience on clinical and self-reported depression

**DOI:** 10.1017/S0033291717003415

**Published:** 2017-11-29

**Authors:** L. B. Navrady, M. J. Adams, S. W. Y. Chan, Major Depressive Disorder Working Group of the Psychiatric Genomics Consortium, S. J. Ritchie, A. M. McIntosh

**Affiliations:** 1 Division of Psychiatry, University of Edinburgh, Royal Edinburgh Hospital, Edinburgh EH10 5HF, UK; 2 Section of Clinical Psychology, University of Edinburgh, Medical Quad, Teviot Place, Edinburgh EH8 9AG, UK; 3 Department of Psychology, University of Edinburgh, 7 George Square, Edinburgh EH8 9JZ, UK; 4 Centre for Cognitive Ageing and Cognitive Epidemiology, University of Edinburgh, 7 George Square, Edinburgh EH8 9JZ, UK; 5 Generation Scotland, Centre for Genetics and Experimental Medicine, Institute for Genetics and Molecular Medicine, University of Edinburgh, Western General Hospital, Crewe Road, Edinburgh EH42XU, UK

**Keywords:** depression, generation Scotland, mediation, moderation, neuroticism, polygenic risk, resilience

## Abstract

**Background:**

Polygenic risk scores (PRS) for depression correlate with depression status and chronicity, and provide causal anchors to identify depressive mechanisms. Neuroticism is phenotypically and genetically positively associated with depression, whereas psychological resilience demonstrates negative phenotypic associations. Whether increased neuroticism and reduced resilience are downstream mediators of genetic risk for depression, and whether they contribute independently to risk remains unknown.

**Methods:**

Moderating and mediating relationships between depression PRS, neuroticism, resilience and both clinical and self-reported depression were examined in a large, population-based cohort, Generation Scotland: Scottish Family Health Study (*N* = 4166), using linear regression and structural equation modelling. Neuroticism and resilience were measured by the Eysenck Personality Scale Short Form Revised and the Brief Resilience Scale, respectively.

**Results:**

PRS for depression was associated with increased likelihood of self-reported and clinical depression. No interaction was found between PRS and neuroticism, or between PRS and resilience. Neuroticism was associated with increased likelihood of self-reported and clinical depression, whereas resilience was associated with reduced risk. Structural equation modelling suggested the association between PRS and self-reported and clinical depression was mediated by neuroticism (43–57%), while resilience mediated the association in the opposite direction (37–40%). For both self-reported and clinical diagnoses, the genetic risk for depression was independently mediated by neuroticism and resilience.

**Conclusions:**

Findings suggest polygenic risk for depression increases vulnerability for self-reported and clinical depression through independent effects on increased neuroticism and reduced psychological resilience. In addition, two partially independent mechanisms – neuroticism and resilience – may form part of the pathway of vulnerability to depression.

## Introduction

Major depressive disorder (MDD) is a pervasive and disabling psychiatric condition characterised by periods of low mood and anhedonia, with an estimated lifetime prevalence of 16% (Levine *et al.* 2014). MDD has substantial public health implications, with research suggesting the disorder increases mortality risk and exacerbates cognitive decline (Reddy, 2010; Ferrari *et al.* 2013). Depression is substantially heritable (Sullivan *et al.* 2000) and has a complex genetic architecture (Liu *et al.* 2011; Schulze *et al.* 2014). Whilst modest progress has been made to understand the heterogeneity of genetic risk factors for MDD (Caspi *et al.* 2003; Duncan & Keller, 2011; Wray *et al.* 2012), genome-wide association studies have indicated that MDD risk is influenced by a large number of common allelic variations of small effect rather than specific susceptibility loci (Lubke *et al.* 2012).

A now-commonplace method applied to examine these genetic influences is polygenic risk scores (PRS) (Demirkan *et al.* 2011) which are used as a measure of ‘genetic liability’ associated with a particular phenotype (Wray *et al.* 2008). PRS are founded on the assumption that whereas genetic variants with very small individual effects may not meet genome-wide significance thresholds (depending on statistical power), their cumulative associations may have a much stronger effect (Wray *et al.* 2007, 2008). Polygenic vulnerabilities have been identified in several psychiatric disorders (Purcell *et al.* 2009; Ripke *et al.* 2013). Specifically to MDD, PRS have been found to correlate with both the status and chronicity of the disorder (Sullivan & MDD Working Group of the Psychiatric Genomics Consortium, 2012; Levine *et al.* 2014). However, to date, PRS typically account for only 1–2% of variance in MDD (Demirkan *et al.* 2011; Cross-Disorder Group of the Psychiatric Genomics Consortium, 2013; Ripke *et al.* 2013) suggesting other factors are also influencing risk for the disorder.

Personality is frequently linked with vulnerability to psychiatric illness (Fanous & Kendler, 2004), with one of the strongest associations being between MDD and neuroticism (Kotov *et al.* 2010). Neuroticism is a partly-heritable personality trait characterised by emotional instability, negative emotional response, and stress sensitivity (Lahey, 2009). Phenotypically, neuroticism is strongly positively associated with MDD both cross-sectionally (Chan *et al.* 2007; Roelofs *et al.* 2008; Navrady *et al.* 2017*a*) and prospectively (Kendler *et al.* 2006; Farmer *et al.* 2008). There is also evidence that neuroticism and depression are strongly genetically correlated (Jardine *et al.* 1984; Kendler *et al.* 1993). For example, evidence from twin studies indicates that neuroticism and MDD share up to two-thirds of their genetic variance (Carey & DiLalla, 1994; Fanous *et al.* 2002; Hettema *et al.* 2006). Furthermore, de Moor and colleagues (de Moor *et al.* 2015) found that neuroticism and MDD can be equally well explained by neuroticism PRS (up to 1.05% variance explained), in addition to being able to predict MDD based on neuroticism PRS alone. As neuroticism is a relatively stable trait (Lahey, 2009), it is hypothesised that it may act as an indirect measure of later risk for MDD, and as such is an important phenotype for MDD genetic studies.

Whereas research into MDD risk has dominated the field, interest in psychological resilience has grown substantially over recent decades (Luthar *et al.* 2006; Russo *et al.* 2012; Southwick & Charney, 2012). Resilience is often described as the positive pole of individual differences in people's susceptibility to MDD, as it is widely observed that not all individuals at risk for the disorder become unwell (Collishaw *et al.* 2007; Alim *et al.* 2008). Resilience has been related to increased positive and reduced negative affect (Smith *et al.* 2010), which suggests potentially different mechanisms for vulnerability and protection (Fredrickson, 2001). Cross-sectionally, it was found that individuals scoring higher on self-reported resilience (using The Connor–Davidson Resilience Scale questionnaire) reported fewer psychiatric symptoms following childhood emotional neglect than did those with lower levels of resilience (Campbell-Sills *et al.* 2006). Using the same measure, higher resilience has been found to mitigate the severity of depressive symptoms in individuals exposed to trauma (Wingo *et al.* 2013). Furthermore, reports suggest that resilience reduces risk for depression in individuals with high genetic loading for the disorder (Wichers *et al.* 2007; Wichers *et al.* 2008; Geschwind *et al.* 2010).

Current research suggests a positive association between neuroticism and MDD; self-reported resilience and depression show a negative association. However, studies often fail to adequately consider how MDD is measured (Adli *et al.* 2006; Cameron *et al.* 2011). Whilst moderate associations between clinical and self-reported measures of MDD suggest the two approaches are interchangeable (Kessler *et al.* 1998; Rush *et al.* 2006), important distinctions between clinical and self-reported depression have been found. Specifically, self-reported and clinical measures of depression have each been found to provide unique information about the disorder not captured by the other (Uher *et al.* 2012) that may help to elucidate underlying mechanisms (Fava *et al.* 1986; Möller, 2000).

**Fig. 1.**
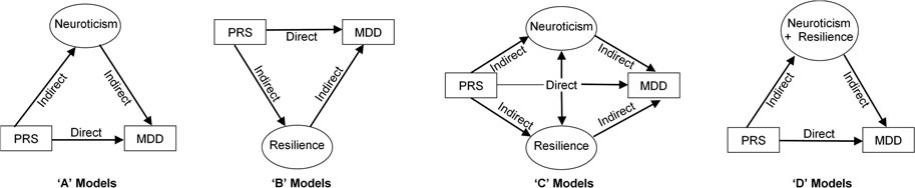
The theoretical mediation models tested in the present study. A first set of models predict clinical MDD status from polygenic risk (PRS) for the disorder (e.g. Model 1A), a second set of models will model self-reported MDD as an outcome (e.g. Model 2A). These models will be conducted with both SCID MDD status representing clinical MDD and CIDI-SF MDD status representing self-reported MDD. ‘A’ models propose that the association between PRS for MDD and clinical and self-reported MDD may be mediated by the latent factor neuroticism. ‘B’ models propose that the association between PRS for depression and clinical and self-reported MDD may be mediated by the latent factor resilience. ‘C’ models propose that, in addition to neuroticism mediating PRS to increase risk for clinical and self-reported MDD, resilience may provide a separate mediating effect to reduce the risk for clinical and self-reported MDD in those genetically liable for the disorder(s). By contrast, ‘D’ models test the assumption that neuroticism and resilience may mediate the link between PRS and clinical and self-reported MDD as one underlying factor; Neuroticism + Resilience. MDD, Major Depressive Disorder; PRS, Polygenic Risk Score; SCID, Structured Clinical Interview for DSM-IV Axis I Disorders; CIDI-SF, Composite International Diagnostic Interview – Short Form.

Here, we report a moderation and a mediation analysis of a large population-based cohort (Generation Scotland: Scottish Family Health Study) who completed both self-reported and clinical measures of MDD. First, in a series of moderation analyses, we investigated whether the association between PRS for MDD and clinical and self-reported depression (Sullivan & MDD Working Group of the Psychiatric Genomics Consortium, 2012; Levine *et al.* 2014) was moderated by neuroticism or resilience. We predicted that neuroticism would be associated with increased likelihood of both clinical and self-reported depression, whilst resilience would associate in the opposite direction, in line with previous findings. Second, using structural equation modelling, we examined if neuroticism mediates the relationship between PRS for MDD and both clinical and self-reported MDD to increase the risk for the disorder, and if resilience would mediate in the opposite direction. The path models we tested are illustrated in Fig. 1.

## Methods

### Participants

Participants were sampled from the Generation Scotland: Scottish Family Health Study (GS:SFHS) – a family-based epidemiological cohort recruited between 2006 and 2011 (Smith *et al.* 2006, 2013). At baseline, participants provided extensive data, including personality measures, a structured interview for clinical MDD diagnosis and DNA extraction. In 2014, GS:SFHS participants were re-contacted and asked to take part in a follow-up assessment of mental health and resilience (Navrady *et al.* 2017*b*), providing a range of questionnaire measures including resilience and self-reported MDD. Full details of the initial recruitment and follow-up have been given elsewhere (Smith *et al*. 2006; 2013; Navrady *et al.* 2017*b*) and in the online Supplementary Materials. This study includes 4166 unrelated individuals (Mean_age_ = 56.01, s.d. = 12.31, *N*_female_ = 2634) with complete data of interest.

GS:SFHS received ethical approval from the NHS Tayside Committee (reference 05/S1401/89 and 14/SS/0039). All participants provided written informed consent.

### Study assessments

DNA was extracted from participants for whole-genome genotyping, the procedures for which have been reported extensively elsewhere (Smith *et al.* 2006; Zeng *et al.* 2016). Genome-wide genotype data were available for all participants in the current study from which PRS were created. Using the genotype data and PRCise software (Euesden *et al.* 2015), PRS were calculated by computing the genome-wide sum of trait-associated alleles across genetic loci, weighted by their effect in an independent genome-wide association study (GWAS). The GWAS summary statistics used for these PRS were those from the large unpublished meta-analysis of MDD from the Psychiatric Genetics Consortium (PGC MDD29; 130 664 MDD cases *v.* 330 470 controls), although GS:SFHS participants were removed from these summary statistics before calculating PRS. Here, we only report findings using a PRS threshold of 0.50 as preliminary analysis indicated this threshold was most predictive of both self-reported and clinical MDD in this sample (see online Supplementary Material).

Neuroticism was assessed using the self-report questionnaire Eysenck Personality Questionnaire Short Form-Revised (EPQ-SF; Eysenck, 1991). The neuroticism subsection of the EPQ-SF consists of 12 ‘Yes/No’ questions (e.g. ‘Are you a worrier?’). Scores range from 0 to 12, with higher scores indicative of higher levels of neuroticism. This scale has been concurrently validated with other quantitative measures of neuroticism (Gow *et al.* 2005) with high reliability (Eysenck *et al.* 1985).

Psychological resilience was assessed using the Brief Resilience Scale (BRS; Smith *et al.* 2008), a self-report questionnaire used to assess an individual's ability to ‘bounce back’ or recover from stress. The BRS consists of six statements (e.g. ‘I usually come through difficult times with little trouble’) answered on a five-point scale from ‘Strongly Disagree’ to ‘Strongly Agree’. After reverse coding of even-numbered questions, a total resilience score was calculated by computing the mean of six questions. The BRS has been found to have a one-factor structure, demonstrating good internal consistency and test–retest reliability (Smith *et al.* 2008).

Participants were screened for a clinical diagnosis of MDD at baseline using the Structured Clinical Interview for DSM-IV Axis I Disorders (SCID; First *et al*. 1997). Diagnosis of MDD followed DSM-IV criteria (American Psychiatric Association, 2003); if either symptom of depressive mood or anhedonia are endorsed, a minimum of four further symptoms must also be endorsed. These symptoms must have lasted nearly all day, every day for a minimum of 2 weeks. Interview procedures and quality control protocol have been described elsewhere (Fernandez-Pujals *et al.* 2015). As the interviews were conducted by a trained researcher SCID MDD status can be used as a proxy for clinical MDD. In this sample, 664 individuals met criteria for clinical MDD (16%), and 3502 were non-MDD cases (84%).

During re-contact, self-reported MDD was assessed using a questionnaire developed by the World Health Organization: the Composite International Diagnostic Interview – Short Form (CIDI-SF; Kessler *et al.* 1998). The CIDI-SF evaluates self-reported MDD according to DSM-IV (American Psychiatric Association, 2003) criteria and employs a stem-branch logic to eliminate individuals who fail to endorse a minimum of four symptoms (in addition to depressed mood and/or anhedonia) with clinical significance. Although the CIDI-SF is a self-report measure of MDD, respondents meeting MDD criteria with the CIDI-SF have been shown to reliably meet full diagnostic criteria with excellent accuracy if given the full version of the questionnaire (Kessler *et al.* 1998). A total of 1068 individuals in the mental health follow-up sample met criteria for self-reported MDD (26%), with 3098 classified as non-MDD cases (74%).

### Analyses

All analyses were conducted using R version 3.2.3 (http://www.R-project.org).

#### Moderation

We performed generalised linear models to examine the moderating associations of both neuroticism and resilience on the relationship between PRS for MDD and clinical and self-reported MDD (SCID and CIDI-SF, respectively). As MDD status is a dichotomous variable, we specified a ‘binary’ family with a logit link function in the analysis. Three moderation models were computed for each MDD category (self-reported and clinical). A basic, first-step model was estimated to examine the validity of the PRS by testing for an association between genetic risk for depression and both clinical and self-reported MDD status. In the second step of the analysis, an interaction model was fitted to estimate the moderating association of neuroticism (total EPQ-SF score) on the contribution of genetic liability to both clinical and self-reported MDD. Another model was then fitted to examine the interaction between PRS and resilience (total BRS score) on susceptibility to clinical and self-reported MDD. Regression coefficients are reported as odds ratios (ORs) with 95% confidence intervals (CIs). The *p* values presented are raw and uncorrected for multiple testing. All continuous variables have been scaled to have a mean of 0 and a standard deviation of 1. As neuroticism and resilience were measured at different time-points, neuroticism was controlled for age at baseline (Age_t1_) and resilience was controlled for age at re-contact (Age_t2_) prior to them entering the moderation models. All models were controlled for four ancestry-informative principal components to take account of possible population stratification; results of these associations are presented in the online Supplementary Material.

#### Mediation

The structural equation modelling package ‘lavaan’ (Rosseel, 2012) was used in R to estimate and compare models of the types shown in Fig. 1. Diagonally Weighted Least Squares (DWLS) estimation was used in all models to account for MDD being a binary variable. The variance of each latent construct was fixed to 1 so as to identify each model. To assess the absolute fit of each model, a range of model-fit indices are reported (MacCallum *et al.* 1996; McQuitty): Root mean square error of approximation (RMSEA; values indicating good fit <0.05), comparative fit index (CFI; values >0.95), and Tucker–Lewis index (TLI; values >0.95). To calculate the percentage mediation in each model we divided the sum of the indirect paths by the total variance explained by the model (Rosseel, 2012). For comparison of one- and two-factor models, we performed chi-squared tests, as an Akaike information criterion (AIC) cannot be computed when using the DWLS method.

As seen in Fig. 1, four mediation models have been produced each for SCID and CIDI-SF MDD status to examine the association between PRS and clinical and self-reported MDD, respectively. ‘A’ models examined the mediating effects of neuroticism (estimated as a latent variable using individual EPQ-SF items) on the relationship between PRS and MDD. The second set of models (‘B’) investigated resilience as a latent variable indicated by individual BRS items as a mediator between PRS and MDD. ‘C’ models examined neuroticism and resilience as two separate latent mediating variables between PRS and MDD. We also examined the phenotypic correlation between these latent variables within the model. Finally, we created a latent variable, named ‘Neuroticism + Resilience’, consisting of all individual EPQ-SF and BRS questionnaire items to determine if one general factor can better explain the relationship between PRS and clinical and self-reported MDD (‘D’ models).

## Results

Descriptive statistics and a correlation matrix are provided in Table 1. As illustrated in Table 1, resilience and neuroticism were moderately negatively correlated (*r* = −0.48, *p* < 0.001). Further demographic information, a full correlation matrix, and the differences and overlap between clinical and self-reported MDD measures in this study are outlined in the online Supplementary Material.

**Table 1.** Correlation Matrix and Descriptive Statistics for baseline age, age at recontact, sex, resilience, neuroticism, clinical and self-reported MDD status

	Age_t1_	Age_t2_	Sex	Resilience	Neuroticism	SCID	CIDI-SF	Mean (s.d.)	*N* (%)
Age_t1_	–							50.28 (12.34)	
Age_t2_	0.99	–						56.01 (12.31)	
Sex (F)	−0.09**	−0.09**	–						2634 (63)
Resilience	0.05	0.05	−0.10**	–				3.52 (0.82)	
Neuroticism	−0.14	−0.14	0.17**	−0.48	–			3.70 (3.17)	
SCID	−0.04**	−0.04**	0.18*	−0.31**	0.36**	–			664 (16)
CIDI-SF	−0.08**	−0.08**	0.24*	−0.35**	0.29**	0.60*	–		1068 (26)

Age_t1_, Age at baseline; Age_t2_, Age at re-contact; Resilience, Total score from the Brief Resilience Scale; Neuroticism, Total score from the Eysenck Personality Questionnaire Short-Form; SCID, Structured Clinical Interview for DSM-IV Axis I Disorders representing clinical MDD; CIDI-SF, Composite International Diagnostic Interview – Short Form representing self-reported MDD.

N.B. All *p* values significant at *p* ⩽ 0.01.

Demographic information for Sex represents the number and percentage of females in this sample. Demographic details for SCID and CIDI-SF represent the number and percentage of participants meeting criteria for clinical and self-reported MDD, respectively.

All coefficients represent Pearson correlations except those denoted by * which represents tetrachoric correlations – resultant from both variables being binary, and those denoted by ** which represent point biserial correlations – resultant from binary and continuous variables.

### Moderation

#### Validity of the MDD PRS

The polygenic risk for MDD was found to be associated with increased likelihood of clinical MDD (see Table 2). A 1 s.d. increase in genetic liability to depression was associated with an increased likelihood of clinical depression by an OR of 1.20 [(95% CIs 1.11–1.31), *p* < 0.001]. Age was found to be associated with clinical MDD, and being female increased clinical MDD likelihood by an OR of 1.72 (95% CIs 1.42–2.07, *p* < 0.001).

**Table 2.** Results of a generalised linear model predicting odds ratios for self-reported and clinical MDD status, p value, upper and lower 95% confidence intervals and the Akaike Information Criterion

MDD Outcome	Variables	Odds ratio	Lower 95% CIs	Upper 95% CIs	*p* Value	AIC
SCID	Age_t1_	0.99	0.99	1.00	0.031	3607.60
	Sex (F)	1.71	1.42	2.07	<0.001	
	PRS	1.20	1.11	1.31	<0.001	
CIDI-SF	Age_t2_	0.99	0.98	0.99	<0.001	4632.80
	Sex (F)	1.94	1.66	2.28	<0.001	
	PRS	1.18	1.10	1.27	<0.001	
SCID	Age _t1_	0.99	0.99	1.00	0.026	3155.90
	Sex (F)	1.33	1.09	1.63	0.005	
	PRS	1.16	1.05	1.29	0.004	
	Neuroticism	2.49	2.28	2.72	<0.001	
	PRS × Neuroticism	0.92	0.84	1.00	0.062	
CIDI-SF	Age_t2_	0.99	0.98	0.99	<0.001	4366.40
	Sex (F)	1.66	1.41	1.95	<0.001	
	PRS	1.13	1.05	1.22	0.002	
	Neuroticism	1.81	1.68	1.95	<0.001	
	PRS × Neuroticism	0.97	0.90	1.04	0.416	
SCID	Age_t1_	0.99	0.99	1.00	0.030	3251.90
	Sex (F)	1.53	1.26	1.86	<0.001	
	PRS	1.17	1.06	1.30	0.002	
	Resilience	0.44	0.40	0.48	<0.001	
	PRS × Resilience	1.06	0.97	1.16	0.211	
CIDI-SF	Age_t2_	0.99	0.98	0.99	<0.001	4156.80
	Sex (F)	1.80	1.52	2.12	<0.001	
	PRS	1.14	1.06	1.24	0.001	
	Resilience	0.43	0.40	0.47	<0.001	
	PRS × Resilience	1.07	0.99	1.17	0.080	

SCID, Structured Clinical Interview for DSM-IV Axis I Disorders, representing clinical MDD; CIDI-SF, Composite International Diagnostic Interview – Short Form, representing self-reported MDD; MDD, Major Depressive Disorder; AIC, Akaike Information Criterion; PRS, Polygenic Risk Score; Age_t1_, Age at the time of baseline; Age_t2_, Age at the time of re-contact.

N.B. Neuroticism has been controlled for Age_t1_ and resilience has been controlled for Age_t2_ before entering the model. Four principal components controlling for population stratification have been adjusted for and are reported in the online Supplementary Material.

Similar results were obtained for self-reported MDD, see Table 2. Specifically, a 1 s.d. increase in polygenic risk for depression increased likelihood of self-reported depression by an OR of 1.18 (95% CIs 1.10–1.27), *p* < 0.001). Age had a small negative association with self-reported MDD, whereas being female increased self-reporting of depression by an OR of 1.94 (95% CIs 1.66–2.28), *p* < 0.001).

#### Interaction between neuroticism and PRS on MDD

No interaction was found between neuroticism and PRS on clinical MDD status [OR 0.92, (95% CI 0.84–1.00), *p* = 0.062], see Table 2. PRS remained associated with clinical MDD [OR 1.16, (95% CIs 1.05–1.29), *p* = 0.004], and neuroticism independently associated with increased likelihood of clinical MDD status [OR 2.49, (95% CI 2.28–2.72), *p* < 0.001].

No interaction was found between neuroticism and PRS on self-reported MDD [OR 0.97, (95% CI 0.90–1.04), *p* = 0.416], see Table 2. PRS remained associated with self-reported depression when co-varying for neuroticism (OR 1.13, (95% CIs 1.05–1.22), *p* = 0.002], and neuroticism also remained strongly independently associated with self-reported MDD status (OR 1.81, (95% CI 1.68–1.95), *p* < 0.001].

#### Interaction between resilience and PRS on MDD

No interaction was found between PRS and resilience in association with clinical MDD [OR 1.04, (95% CI 0.95–1.14), *p* = 0.373]; see Table 2. The main effect of PRS was associated with clinical MDD [OR 1.19, (95% CI 1.08–1.32), *p* = 0.001]. A strong inverse relationship was found between resilience and clinical depression [OR 0.44, (95% CI 0.40, 0.48), *p* < 0.001].

No interaction was found between PRS and resilience on self-reported MDD [OR 1.06, (95% CI 0.97–1.16), *p* = 0.211; see Table 2]. Whereas the main effect of PRS was associated with increased likelihood of self-reported depression [OR 1.17, (95% CI 1.06–1.30), *p* = 0.002], resilience was found to be associated with a reduction in self-reported MDD [OR 0.44, (95% CI 0.40–0.48), *p* < 0.001].

### Mediation

#### Mediation of neuroticism

Model 1A showed no direct association between PRS and clinical MDD status (*β* = 0.04, *p* = 0.077), although this pathway was estimated to explain 4.4% of the variance. The path from PRS to neuroticism demonstrated a small positive association (*β* = 0.07, *p* < 0.001). A larger association between neuroticism and clinical MDD was found (*β* = 0.87, *p* < 0.001). This indirect pathway explained 5.8% of the variance. As shown in Table 3, Model 1A had a good fit to the data, and suggested that 57% of the association of genetic liability for depression on clinical MDD was mediated by neuroticism.

**Table 3.** Fit statistics for the mediation models tested with both clinical and self-reported MDD status as an outcome

Model	MDD outcome	Model description	df	χ^2^	CFI	TLI	Null RMSEA	RMSEA	RMSEA Lower CI	RMSEA Upper CI
1A	SCID	Neuroticism as a mediator	142	165.97	0.977	0.972	0.038^a^	0.006	0.000	0.010
2A	CIDI-SF		155	168.93	0.986	0.983	0.036^a^	0.005	0.000	0.009
1B	SCID	Resilience as a mediator	56	159.05	0.992	0.989	0.201	0.021	0.017	0.025
2B	CIDI-SF		49	152.31	0.992	0.989	0.214	0.022	0.019	0.027
1C	SCID	Neuroticism & resilience as separate mediators	281	336.70	0.996	0.996	0.107^a^	0.007	0.003	0.010
2C	CIDI-SF		281	336.62	0.997	0.996	0.109^a^	0.007	0.003	0.010
1D	SCID	Neuroticism & resilience as one underlying mediating factor: Neuroticism + Resilience	289	637.17	0.978	0.975	0.107^a^	0.017	0.015	0.019
2D	CIDI-SF		289	596.99	0.981	0.978	0.109^a^	0.016	0.014	0.018

SCID, Structured Clinical Interview for DSM-IV Axis I Disorders, representing clinical MDD; CIDI-SF, Composite International Diagnostic Interview – Short Form, representing self-reported MDD; MDD, Major Depressive Disorder; RMSEA, root-mean-square error of approximation; CFI, comparative fit index; TLI, Tucker–Lewis index.

a TLI and other incremental fit indices may not be that informative, because the RMSEA of the baseline model is lower than 0.158 (Kenny *et al.* 2015).

In Model 2A, the direct path between PRS and self-reported MDD (*β* = 0.06, *p* = 0.013), was estimated to explain 5.5% of the variance. A small association between PRS and neuroticism was found (*β* = 0.06, *p* *<* 0.001), whilst the path from neuroticism to self-reported MDD showed a much stronger association (*β* = 0.65, *p* < 0.001). Together, this indirect pathway explained 4.2% of the variance. As shown in Table 3, Model 2A had a good fit to the data. This model suggested that 43% of the association of genetic liability for depression with self-reported MDD was mediated by neuroticism.

#### Mediation of resilience

In Model 1B, a direct association between PRS and clinical MDD was found (*β* = 0.06, *p* = 0.008, explaining 6.4% of the variance). However, a small negative association between PRS and resilience (*β* = −0.07, *p* < 0.001), and a larger negative association from resilience to clinical MDD (*β* = −0.58, *p* < 0.001), explained 3.8% of the variance. Model 1B's fit to the data was also good (see Table 3), and suggested that 37% of the association between PRS and clinical MDD was mediated by resilience.

A direct association between PRS and self-reported MDD was found in Model 2B (*β* = 0.06, *p* = 0.008), which was estimated to explain 5.8% of the variance. A small, negative association between PRS and resilience was found (*β* = −0.07, *p* < 0.001), in addition to a larger negative association between resilience and self-reported MDD (*β* = −0.60, *p* < 0.001), which together explained 3.9% of the variance. Model 2B's fit to the data was also good (see Table 3), and suggested that 40% of the association between PRS and self-reported MDD was mediated by resilience.

#### Two-factor mediation

**Fig. 2.**
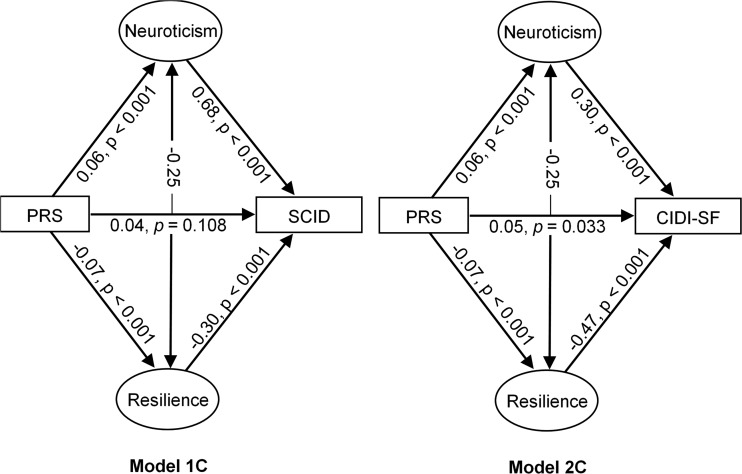
Path diagram of Models 1C and 2C, which include a direct bath between PRS and MDD status, an indirect path through neuroticism and an indirect path through resilience. Model 1C denotes SCID as the MDD outcome, representing clinical depression. Model 2C denotes CIDI-SF as the MDD outcome, representing self-reported depression. Values are standardised path coefficients. All endogenous variables have been adjusted for population stratification, sex and the age at which the variable was measured. PRS, Polygenic Risk Score: MDD, Major Depressive Disorder; SCID, Structured Clinical Interview for DSM-IV Axis I Disorders; CIDI-SF, Composite International Diagnostic Interview – Short Form.

As shown in Fig. 2, Model 1C found no association between PRS and clinical MDD was found (*β* = 0.04, *p* = 0.108), although this direct path was estimated to explain 3.9% of the variance. Whilst a small positive association was found between PRS and neuroticism (*β* = 0.06, *p* < 0.001), a small inverse relationship was found between PRS and resilience (*β* = −0.07, *p* < 0.001). A positive association was found between neuroticism and clinical MDD (*β* = 0.68, *p* < 0.001), whereas a negative association was found between resilience and clinical depression (*β* = −0.30, *p* < 0.001). Neuroticism and resilience were moderately negatively correlated (*r* = −0.25). Table 3 indicates that Model 1C had a good fit to the data. Approximately 61% of the association between PRS and clinical MDD was found to be mediated by neuroticism and resilience, as two separate factors.

In Model 2C, we examined the mediating associations of both neuroticism and resilience as separate constructs between PRS and self-reported MDD, within the same model. As shown in Fig. 2, the direct association between PRS and CIDI-SF (*β* = 0.05, *p* = 0.033), was estimated to explain 4.7% of the variance. A small positive association was found between PRS and neuroticism (*β* = 0.06, *p* < 0.001), and an inverse relationship found between PRS and resilience (*β* = −0.07, *p* < 0.001). The same direction of association was evident in the path between neuroticism and self-reported MDD (*β* = 0.30, *p* < 0.001) and between resilience and self-reported depression (*β* = −0.47, *p* < 0.001). Neuroticism and resilience were found to be negatively correlated (*r* = −0.25). As shown in Table 3, Model 2C had a good fit to the data, with neuroticism and resilience as two separate factors explaining approximately 52% of the association between PRS and self-reported MDD.

#### Neuroticism and resilience as one underlying factor

Model 1D examined if neuroticism and resilience reflect opposite ends of the same trait by creating a latent variable (Neuroticism + Resilience) comprising of all the individual item responses from both the EPQ-SF and the BRS. A small association between PRS and clinical MDD was found (*β* = 0.06, *p* = 0.023), explaining 6% of the variance. An association between PRS to Neuroticism + Resilience was found (*β* = 0.04, *p* < 0.001) in addition to a positive association between Neuroticism + Resilience and clinical MDD (*β* = 1.19, *p* < 0.001). In total, Model 1D explained 47% of the mediation between PRS and clinical MDD. As shown in Table 3, Model 1C appears to fit the data much better than does Model 1D χ^2^(8) = 300.48, *p* < 0.001, suggesting that neuroticism and resilience reflected two partially separate constructs independently mediating the relationship between PRS for MDD and clinical depression.

Model 2D also investigated whether one underlying factor can better explain the mediation of the PRS-depression relation by neuroticism and resilience. A small association was found between PRS and self-reported MDD (*β* = 0.05, *p* = 0.025) which was estimated to explain 4.9% of the variance. A small association between PRS and Neuroticism + Resilience was found (*β* = 0.04, *p* < 0.001) in addition to a positive association between Neuroticism + Resilience and self-reported MDD (*β* = 1.21, *p* < 0.001). Together, these indirect paths explained 4.8% of the variance, whilst the model itself explained 49% of the mediation between PRS and self-reported MDD. Model 2D's fit to the data was significantly poorer than that of Model 2C (see Table 3), χ^2^(8) = 260.37, *p* < 0.001. This suggests neuroticism and resilience should be considered partially independent constructs associated with different mediating mechanisms in the association between genetic liability for MDD and self-reported depression.

## Discussion

Here, we report the first study investigating the moderating and mediating associations of neuroticism and resilience on genetic liability for MDD on both clinical and self-reported depression in a large cohort of individuals. Our results suggest that polygenic risk for MDD is associated with an increased likelihood of both clinical and self-reported depression, replicating previous findings (Sullivan & MDD Working Group of the Psychiatric Genomics Consortium, 2012; Levine *et al.* 2014). Consistent with existing literature (Farmer *et al.* 2008; Roelofs *et al.* 2008; Navrady *et al.* 2017*a*), neuroticism is associated with increased likelihood of both clinical and self-reported MDD, whereas resilience was found to associate in the opposite direction (Geschwind *et al.* 2010; Wingo *et al.* 2013). Overall, our moderation analyses demonstrate an association whereby both genetic liability for MDD and neuroticism increases the likelihood of both clinical and self-reported depression, independently, whereas resilience associates with the reduced likelihood for both clinical and self-reported depression, even after adjusting for genetic vulnerability. However, neuroticism and resilience did not show a multiplicative relation with the PRS, boosting and reducing the size of its association with depression, respectively. Structural equation modelling of this data suggests that genetic liability for MDD is largely mediated by neuroticism to increase the risk for clinical and self-reported depression, whilst resilience mediates PRS to mitigate risk for both clinical and self-reported MDD. Results from this study demonstrate that neuroticism and resilience independently mediate the effects of genetic risk on depression, for both self-reported and clinical measures of MDD.

Whereas the results from our moderation analyses results are consistent with those previously found for PRS (Levine *et al.* 2014), neuroticism (de Moor *et al.* 2015) and resilience (Geschwind *et al.* 2010), our meditational analyses reported novel findings. Consistent with the possibility that polygenic genetic differences shape susceptibility to MDD, our findings further suggest that this relationship is driven by two partially separate mediating mechanisms; one in which neuroticism increases the risk for both clinical and self-reported MDD, the other in which resilience reduces the same risk. Evidence for neuroticism and resilience being partially independent mechanisms comes from our finding that the two measures are not perfectly correlated (*r* = −0.48), in addition to our structural equation models which demonstrate two separate associations.

It is possible that the meditational associations of neuroticism and resilience can be explained by the role of positive and negative emotions. It is well documented that neuroticism is characterised by a range of negative emotions highly associated with MDD (Chan *et al.* 2007; Navrady *et al.* 2017*a*). Although resilience has received less empirical attention, researchers have hypothesised that resilience is characterised by positive emotionality (Wolin & Wolin, 1993; Block & Kremen, 1996; Masten, 2001) which over time provide individuals with an enduring capacity to ‘bounce back’ when MDD would otherwise be expected (Lazarus, 1993; Masten, 2001). Indeed, Fredrickson (2001) has developed the ‘broaden-and-build’ hypothesis of positive emotions which posits that whereas negative emotions narrow an individual's cognitive biases to increase the likelihood of depressive symptoms, positive emotions broaden one's thought–action repertoires to navigate away from the disorder (Fredrickson, 2004). In the present study, PRS for MDD increased vulnerability to both clinical and self-reported depression. This relationship is mediated and increased by neuroticism as the negative emotions it elicits are congruent with the disorder. Resilience may mediate and ameliorate the relationship between PRS for depression and MDD by promoting habituation to stressors, encouraging efficacious coping behaviours and prompting cognitive reappraisal away from depressive mood states (Buhrmester *et al.* 2011; Amstadter *et al.* 2016). These findings may have clinical applications insofar that therapeutic interventions for MDD may benefit from focusing on positive emotions to facilitate recovery and resilience rather than exclusively focused on alleviating psychiatric symptoms (Fredrickson & Joiner, 2002).

Resilient individuals are believed to ‘bounce back’ from adversity quickly and efficiently, akin to the way a spring stretches but still returns to its original form (Lazarus, 1993). Current resilience measures often fail to assess the concept across the lifespan or recognise that risk or adversity is an essential element of resilience (Windle *et al.* 2011). Although the BRS assumes a trait-based conceptualisation of resilience, the measure is framed in regard to negative events (Smith *et al.* 2008), which frequently contribute to the onset of the disorder. Moreover, the inclusion of PRS in our analysis provides a measure of MDD risk that precede the outcome. PRS provide a causal anchor for our mediation analyses as they are a biological measure not subject to reverse causality. Whilst the use of genetic factors is unusual in structural equation modelling, they are helpful within this study as we can be more certain of the causal path directions, and as such, this is not just a correlational analysis. However, a replication would be beneficial. Furthermore, additional work should fully elucidate the concept of resilience, as wide discrepancies exist in its definition and measurement (Bonanno *et al.* 2015). We argue that future research needs to assess resilience across the lifespan to fully understand the processes and mechanisms that underlie it, and how it associates with depression.

Some limitations to this study warrant mention. Firstly, our measures of MDD were taken at different time points. Although our results were robust across the two measures of MDD, we must note the difference in prevalence rates between the clinical (16%) and self-reported (26%) measures. This may be due to a sampling bias at re-contact in which participants with mental health problems were more likely to take part in a study specifically aimed to investigate mental health. Although it has been argued that structured clinical interviews have better psychometric properties than self-report measures of MDD (Ekselius *et al.* 1994), research does suggest that the diagnostic classifications obtained using measures such as the CIDI-SF accurately reflect those made using the SCID (Kessler *et al.* 1998). Whilst clinical and self-reported measures have been found to provide unique information on MDD due to disproportionate weighting of symptoms within each measure (Uher *et al.* 2012), it is widely reported that they each correlate highly when measuring the presence or absence of MDD rather than the severity of symptoms (Fava *et al.* 1986). For this reason, we believe that the use of a self-reported and clinical measure of MDD is advantageous, despite some limitations. In addition, the concept of resilience was entirely self-reported; there is no consensus on how to measure resilience, and other measures (e.g. the off-diagonal method used by van Harmelen *et al*. 2017) may have produced different results. It is also possible that MDD and neuroticism may influence the recall of experienced events, and that correlations between these variables, and resilience, may be introduced as a result. However, despite this potential limitation, neuroticism has been demonstrated to be a relatively stable trait in many previous studies and the use of genetic PRS scores – which must come causally prior to behaviours – provides an anchor for a study that much previous research does not have available. A final limitation of this study pertains to the differences in time between the baseline and re-contact. There is disparity among participants in regards to the time period between their baseline testing and re-contact, with some participants having a longer follow-up period than others. As a result, some participants might have experienced more negative life events, thus increasing their propensity for MDD.

In conclusion, this study suggests that polygenic risk for MDD increases the risk for both clinical and self-reported depression through independent effects on increasing neuroticism and reducing resilience. This study suggests that two partially separate mechanisms – neuroticism and resilience – influence vulnerability and protection to MDD.

## Supplementary material

For supplementary material accompanying this paper visit http://dx.doi.org/10.1017/S0033291717003415. click here to view supplementary material
