# Supplementary material for: Genetic risk of major depressive disorder: the moderating and mediating effects of neuroticism and psychological resilience on clinical and self-reported depression
Source: Psychol Med. 2017 Nov 29;48(11):1890–9. doi: 10.1017/S0033291717003415 (PMC6088772; doi:10.1017/S0033291717003415)
Supplement: Supplementary file 1 [file S0033291717003415sup001.pdf]

Genetic risk of Major Depressive Disorder: the moderating and mediating effects of neuroticism and psychological resilience on clinical and self-reported depression

Navrady, LB., Adams, MJ., Chan, SWY., Major Depressive Disorder Working Group of the Psychiatric Genomics Consortium, Ritchie, SJ., McIntosh, AM.

Supplementary Material

Major Depressive Disorder Working Group of the Psychiatric Genomics Consortium

Naomi R Wray* [1, 2]
Stephan Ripke* [3, 4, 5]
Manuel Mattheisen* [6, 7, 8, 9]
Maciej Trzaskowski* [1]
Enda M Byrne [1]
Abdel Abdellaoui [10]
Mark J Adams [11]
Esben Agerbo [9, 12, 13]
Tracy M Air [14]
Till F M Andlauer [15, 16]
Silviu-Alin Bacanu [17]
Marie Bækvad-Hansen [9, 18]
Aartjan T F Beekman [19]
Tim B Bigdeli [17, 20]
Elisabeth B Binder [15, 21]
Douglas H R Blackwood [11]
Julien Bryois 22
Henriette N Buttenschøn [8, 9, 23]
Jonas Bybjerg-Grauholm [9, 18]
Na Cai [24, 25]
Enrique Castelao [26]
Jane Hvarregaard Christensen [7, 8, 9]
Toni-Kim Clarke [11]
Jonathan R I Coleman [27]
Lucía Colodro-Conde [28]
Baptiste Couvy-Duchesne [29, 30]
Nick Craddock [31]
Gregory E Crawford [32, 33]
Gail Davies [34]
Ian J Deary [34]
Franziska Degenhardt [35, 36]
Eske M Derks [28]
Nese Direk [37, 38]
Conor V Dolan [10]
Erin C Dunn [39, 40, 41]
Thalia C Eley [27]
Valentina Escott-Price [42]
Farnush Farhadi Hassan Kiadeh [43]
Hilary K Finucane [44, 45]
Andreas J Forstner [35, 36, 46, 47]
Josef Frank [48]
Héléna A Gaspar [27]
Michael Gill [49]
Fernando S Goes [50]
Scott D Gordon [51]
Jakob Grove [7, 8, 9, 52]
Lynsey S Hall [11, 53]
Christine Søholm Hansen [9, 18]
Thomas F Hansen [54, 55, 56]
Stefan Herms [35, 36, 47]
Ian B Hickie [57]
Per Hoffmann [35, 36, 47]
Georg Homuth [58]
Carsten Horn [59]
Jouke-Jan Hottenga [10]
David M Hougaard [9, 18]
Marcus Ising [60]
Rick Jansen [19, 19]
Eric Jorgenson [61]
James A Knowles [62]
Isaac S Kohane [63, 64, 65]
Julia Kraft [4]
Warren W. Kretzschmar [66]
Jesper Krogh [67]
Zoltán Kutalik [68, 69]
Yihan Li [66]
Penelope A Lind [28]
Donald J MacIntyre [70, 71]
Dean F MacKinnon [50]
Robert M Maier [2]
Wolfgang Maier [72]
Jonathan Marchini [73]
Hamdi Mbarek [10]
Patrick McGrath [74]
Peter McGuffin [27]
Sarah E Medland [28]
Divya Mehta [2, 75]
Christel M Middeldorp [10, 76, 77]
Evelin Mihailov [78]
Yuri Milaneschi [19, 19]
Lili Milani [78]
Francis M Mondimore [50]
Grant W Montgomery [1]
Sara Mostafavi [79, 80]
Niamh Mullins [27]
Matthias Nauck [81, 82]
Bernard Ng [80]
Michel G Nivard [10]
Dale R Nyholt [83]
Paul F O'Reilly [27]
Hogni Oskarsson [84]
Michael J Owen [85]
Jodie N Painter [28]
Carsten Bøcker Pedersen [9, 12, 13]
Marianne Giørtz Pedersen [9, 12, 13]
Roseann E. Peterson [17, 86]
Erik Pettersson [22]
Wouter J Peyrot [19]
Giorgio Pistis [26]
Danielle Posthuma [87, 88]
Jorge A Quiroz [89]
Per Qvist [7, 8, 9]
John P Rice [90]
Brien P. Riley [17]
Margarita Rivera [27, 91]
Saira Saeed Mirza [37]
Robert Schoevers [92]
Eva C Schulte [93, 94]
Ling Shen [61]
Jianxin Shi [95]
Stanley I Shyn [96]
Engilbert Sigurdsson [97]
Grant C B Sinnamon [98]
Johannes H Smit [19]
Daniel J Smith [99]
Hreinn Stefansson [100]
Stacy Steinberg [100]
Fabian Streit [48]
Jana Strohmaier [48]
Katherine E Tansey [101]
Henning Teismann [102]
Alexander Teumer [103]
Wesley Thompson [9, 55, 104, 105]
Pippa A Thomson [106]
Thorgeir E Thorgeirsson [100]
Matthew Traylor [107]
Jens Treutlein [48]
Vassily Trubetskoy [4]
André G Uitterlinden [108]
Daniel Umbricht [109]
Sandra Van der Auwera [110]
Albert M van Hemert [111]
Alexander Viktorin [22]
Peter M Visscher [1, 2]
Yunpeng Wang [9, 55, 105]
Bradley T. Webb [112]
Shantel Marie Weinsheimer [9, 55]
Jürgen Wellmann [102]
Gonneke Willemsen [10]
Stephanie H Witt [48]
Yang Wu [1]
Hualin S Xi [113]
Jian Yang 2, [114]
Futao Zhang [1]
Volker Arolt [115]

Bernhard T Baune [14]
Klaus Berger [102]
Dorret I Boomsma [10]
Sven Cichon [35, 47, 116, 117]
Udo Dannlowski [115]
EJC de Geus [10, 118]
J Raymond DePaulo [50]
Enrico Domenici [119]
Katharina Domschke [120]
Tõnu Esko [5, 78]
Hans J Grabe [110]
Steven P Hamilton [121]
Caroline Hayward [122]
Andrew C Heath [90]
Kenneth S Kendler [17]
Stefan Kloiber [60, 123, 124]
Glyn Lewis [125]
Qingqin S Li [126]
Susanne Lucae [60]
Pamela AF Madden [90]
Patrik K Magnusson [22]
Nicholas G Martin [51]
Andrew M McIntosh [11, 34]
Andres Metspalu [78, 127]
Ole Mors [9, 128]
Preben Bo Mortensen [8, 9, 12, 13]
Bertram Müller-Myhsok [15, 16, 129]
Merete Nordentoft [9, 130]
Markus M Nöthen [35, 36]
Michael C O'Donovan [85]
Sara A Paciga [131]
Nancy L Pedersen [22]
Brenda WJH Penninx [19]
Roy H Perlis [39, 132]
David J Porteous [106]
James B Potash [133]
Martin Preisig [26]
Marcella Rietschel [48]
Catherine Schaefer [61]
Thomas G Schulze [48, 94, 134, 135, 136]
Jordan W Smoller [39, 40, 41]
Kari Stefansson [100, 137]
Henning Tiemeier [37, 138, 139]
Rudolf Uher [140]
Henry Völzke [103]
Myrna M Weissman [74, 141]
Thomas Werge [9, 55, 142]
Cathryn M Lewis* [27, 143]
Douglas F Levinson* [144]
Gerome Breen* [27, 145]
Anders D Børglum* [7, 8, 9]
Patrick F Sullivan* [22, 146, 147]

1. Institute for Molecular Bioscience, The University of Queensland, Brisbane, QLD, AU
2. Queensland Brain Institute, The University of Queensland, Brisbane, QLD, AU
3. Analytic and Translational Genetics Unit, Massachusetts General Hospital, Boston, MA, US
4. Department of Psychiatry and Psychotherapy, Universitätsmedizin Berlin Campus Charité Mitte, Berlin, DE
5. Medical and Population Genetics, Broad Institute, Cambridge, MA, US
6. Centre for Psychiatry Research, Department of Clinical Neuroscience, Karolinska Institutet, Stockholm, SE
7. Department of Biomedicine, Aarhus University, Aarhus, DK
8. iSEQ, Centre for Integrative Sequencing, Aarhus University, Aarhus, DK
9. iPSYCH, The Lundbeck Foundation Initiative for Integrative Psychiatric Research, DK
10. Dept of Biological Psychology & EMGO+ Institute for Health and Care Research, Vrije Universiteit Amsterdam, Amsterdam, NL
11. Division of Psychiatry, University of Edinburgh, Edinburgh, GB
12. Centre for Integrated Register-based Research, Aarhus University, Aarhus, DK
13. National Centre for Register-Based Research, Aarhus University, Aarhus, DK
14. Discipline of Psychiatry, University of Adelaide, Adelaide, SA, AU
15. Department of Translational Research in Psychiatry, Max Planck Institute of Psychiatry, Munich, DE
16. Munich Cluster for Systems Neurology (SyNergy), Munich, DE
17. Department of Psychiatry, Virginia Commonwealth University, Richmond, VA, US
18. Center for Neonatal Screening, Department for Congenital Disorders, Statens Serum Institut, Copenhagen, DK
19. Department of Psychiatry, Vrije Universiteit Medical Center and GGZ inGeest, Amsterdam, NL
20. Virginia Institute for Psychiatric and Behavior Genetics, Richmond, VA, US
21. Department of Psychiatry and Behavioral Sciences, Emory University School of Medicine, Atlanta, GA, US
22. Department of Medical Epidemiology and Biostatistics, Karolinska Institutet, Stockholm, SE
23. Department of Clinical Medicine, Translational Neuropsychiatry Unit, Aarhus University, Aarhus, DK
24. Human Genetics, Wellcome Trust Sanger Institute, Cambridge, GB
25. Statistical genomics and systems genetics, European Bioinformatics Institute (EMBL-EBI), Cambridge, GB
26. Department of Psychiatry, University Hospital of Lausanne, Prilly, Vaud, CH
27. MRC Social Genetic and Developmental Psychiatry Centre, King's College London, London, GB
28. Genetics and Computational Biology, QIMR Berghofer Medical Research Institute, Herston, QLD, AU
29. Centre for Advanced Imaging, The University of Queensland, Saint Lucia, QLD, AU
30. Queensland Brain Institute, The University of Queensland, Saint Lucia, QLD, AU
31. Psychological Medicine, Cardiff University, Cardiff, GB

32. Center for Genomic and Computational Biology, Duke University, Durham, NC, US
33. Department of Pediatrics, Division of Medical Genetics, Duke University, Durham, NC, US
34. Centre for Cognitive Ageing and Cognitive Epidemiology, University of Edinburgh, Edinburgh, GB
35. Institute of Human Genetics, University of Bonn, Bonn, DE
36. Life&Brain Center, Department of Genomics, University of Bonn, Bonn, DE
37. Epidemiology, Erasmus MC, Rotterdam, Zuid-Holland, NL
38. Psychiatry, Dokuz Eylul University School Of Medicine, Izmir, TR
39. Department of Psychiatry, Massachusetts General Hospital, Boston, MA, US
40. Psychiatric and Neurodevelopmental Genetics Unit (PNGU), Massachusetts General Hospital, Boston, MA, US
41. Stanley Center for Psychiatric Research, Broad Institute, Cambridge, MA, US
42. Neuroscience and Mental Health, Cardiff University, Cardiff, GB
43. Bioinformatics, University of British Columbia, Vancouver, BC, CA
44. Department of Epidemiology, Harvard T.H. Chan School of Public Health, Boston, MA, US
45. Department of Mathematics, Massachusetts Institute of Technology, Cambridge, MA, US
46. Department of Psychiatry (UPK), University of Basel, Basel, CH
47. Human Genomics Research Group, Department of Biomedicine, University of Basel, Basel, CH
48. Department of Genetic Epidemiology in Psychiatry, Central Institute of Mental Health, Medical Faculty Mannheim, Heidelberg University, Mannheim, Baden-Württemberg, DE
49. Department of Psychiatry, Trinity College Dublin, Dublin, IE
50. Psychiatry & Behavioral Sciences, Johns Hopkins University, Baltimore, MD, US
51. Genetics and Computational Biology, QIMR Berghofer Medical Research Institute, Brisbane, QLD, AU
52. Bioinformatics Research Centre, Aarhus University, Aarhus, DK
53. Institute of Genetic Medicine, Newcastle University, Newcastle upon Tyne, GB
54. Danish Headache Centre, Department of Neurology, Rigshospitalet, Glostrup, DK
55. Institute of Biological Psychiatry, Mental Health Center Sct. Hans, Mental Health Services Capital Region of Denmark, Copenhagen, DK
56. iPSYCH, The Lundbeck Foundation Initiative for Psychiatric Research, Copenhagen, DK
57. Brain and Mind Centre, University of Sydney, Sydney, NSW, AU
58. Interfaculty Institute for Genetics and Functional Genomics, Department of Functional Genomics, University Medicine and Ernst Moritz Arndt University Greifswald, Greifswald, Mecklenburg-Vorpommern, DE
59. Roche Pharmaceutical Research and Early Development, Pharmaceutical Sciences, Roche Innovation Center Basel, F. Hoffmann-La Roche Ltd, Basel, CH
60. Max Planck Institute of Psychiatry, Munich, DE
61. Division of Research, Kaiser Permanente Northern California, Oakland, CA, US
62. Psychiatry & The Behavioral Sciences, University of Southern California, Los Angeles, CA, US
63. Department of Biomedical Informatics, Harvard Medical School, Boston, MA, US
64. Department of Medicine, Brigham and Women's Hospital, Boston, MA, US
65. Informatics Program, Boston Children's Hospital, Boston, MA, US
66. Wellcome Trust Centre for Human Genetics, University of Oxford, Oxford, GB
67. Department of Endocrinology at Herlev University Hospital, University of Copenhagen, Copenhagen, DK
68. Institute of Social and Preventive Medicine (IUMSP), University Hospital of Lausanne, Lausanne, VD, CH
69. Swiss Institute of Bioinformatics, Lausanne, VD, CH
70. Division of Psychiatry, Centre for Clinical Brain Sciences, University of Edinburgh, Edinburgh, GB
71. Mental Health, NHS 24, Glasgow, GB
72. Department of Psychiatry and Psychotherapy, University of Bonn, Bonn, DE
73. Statistics, University of Oxford, Oxford, GB
74. Psychiatry, Columbia University College of Physicians and Surgeons, New York, NY, US
75. School of Psychology and Counseling, Queensland University of Technology, Brisbane, QLD, AU
76. Child and Youth Mental Health Service, Children's Health Queensland Hospital and Health Service, South Brisbane, QLD, AU
77. Child Health Research Centre, University of Queensland, Brisbane, QLD, AU

78. Estonian Genome Center, University of Tartu, Tartu, EE
79. Medical Genetics, University of British Columbia, Vancouver, BC, CA
80. Statistics, University of British Columbia, Vancouver, BC, CA
81. DZHK (German Centre for Cardiovascular Research), Partner Site Greifswald, University Medicine, University Medicine Greifswald, Greifswald, Mecklenburg-Vorpommern, DE
82. Institute of Clinical Chemistry and Laboratory Medicine, University Medicine Greifswald, Greifswald, Mecklenburg-Vorpommern, DE
83. Institute of Health and Biomedical Innovation, Queensland University of Technology, Brisbane, QLD, AU
84. Humus, Reykjavik, IS
85. MRC Centre for Neuropsychiatric Genetics and Genomics, Cardiff University, Cardiff, GB
86. Virginia Institute for Psychiatric & Behavioral Genetics, Virginia Commonwealth University, Richmond, VA, US
87. Clinical Genetics, Vrije Universiteit Medical Center, Amsterdam, NL
88. Complex Trait Genetics, Vrije Universiteit Amsterdam, Amsterdam, NL
89. Solid Biosciences, Boston, MA, US
90. Department of Psychiatry, Washington University in Saint Louis School of Medicine, Saint Louis, MO, US
91. Department of Biochemistry and Molecular Biology II, Institute of Neurosciences, Center for Biomedical Research, University of Granada, Granada, ES
92. Department of Psychiatry, University of Groningen, University Medical Center Groningen, Groningen, NL
93. Department of Psychiatry and Psychotherapy, Medical Center of the University of Munich, Campus Innenstadt, Munich, DE
94. Institute of Psychiatric Phenomics and Genomics (IPPG), Medical Center of the University of Munich, Campus Innenstadt, Munich, DE
95. Division of Cancer Epidemiology and Genetics, National Cancer Institute, Bethesda, MD, US
96. Behavioral Health Services, Kaiser Permanente Washington, Seattle, WA, US
97. Faculty of Medicine, Department of Psychiatry, University of Iceland, Reykjavik, IS
98. School of Medicine and Dentistry, James Cook University, Townsville, QLD, AU
99. Institute of Health and Wellbeing, University of Glasgow, Glasgow, GB
100. deCODE Genetics / Amgen, Reykjavik, IS
101. College of Biomedical and Life Sciences, Cardiff University, Cardiff, GB
102. Institute of Epidemiology and Social Medicine, University of Münster, Münster, Nordrhein-Westfalen, DE
103. Institute for Community Medicine, University Medicine Greifswald, Greifswald, Mecklenburg-Vorpommern, DE
104. Department of Psychiatry, University of California, San Diego, San Diego, CA, US
105. KG Jebsen Centre for Psychosis Research, Norway Division of Mental Health and Addiction, Oslo University Hospital, Oslo, NO
106. Medical Genetics Section, CGEM, IGMM, University of Edinburgh, Edinburgh, GB
107. Clinical Neurosciences, University of Cambridge, Cambridge, GB
108. Internal Medicine, Erasmus MC, Rotterdam, Zuid-Holland, NL
109. Roche Pharmaceutical Research and Early Development, Neuroscience, Ophthalmology and Rare Diseases Discovery & Translational Medicine Area, Roche Innovation Center Basel, F. Hoffmann-La Roche Ltd, Basel, CH
110. Department of Psychiatry and Psychotherapy, University Medicine Greifswald, Greifswald, Mecklenburg-Vorpommern, DE
111. Department of Psychiatry, Leiden University Medical Center, Leiden, NL
112. Virginia Institute of Psychiatric & Behavioral Genetics, Virginia Commonwealth University, Richmond, VA, US
113. Computational Sciences Center of Emphasis, Pfizer Global Research and Development, Cambridge, MA, US
114. Institute for Molecular Bioscience; Queensland Brain Institute, The University of Queensland, Brisbane, QLD, AU
115. Department of Psychiatry, University of Münster, Münster, Nordrhein-Westfalen, DE
116. Institute of Medical Genetics and Pathology, University Hospital Basel, University of Basel, Basel, CH

117. Institute of Neuroscience and Medicine (INM-1), Research Center Juelich, Juelich, DE
118. Amsterdam Public Health Institute, Vrije Universiteit Medical Center, Amsterdam, NL
119. Centre for Integrative Biology, Università degli Studi di Trento, Trento, Trentino-Alto Adige, IT
120. Department of Psychiatry and Psychotherapy, Medical Center, University of Freiburg, Faculty of Medicine, University of Freiburg, Freiburg, DE
121. Psychiatry, Kaiser Permanente Northern California, San Francisco, CA, US
122. Medical Research Council Human Genetics Unit, Institute of Genetics and Molecular Medicine, University of Edinburgh, Edinburgh, GB
123. Department of Psychiatry, University of Toronto, Toronto, ON, CA
124. Centre for Addiction and Mental Health, Toronto, ON, CA
125. Division of Psychiatry, University College London, London, GB
126. Neuroscience Therapeutic Area, Janssen Research and Development, LLC, Titusville, NJ, US
127. Institute of Molecular and Cell Biology, University of Tartu, Tartu, EE
128. Psychosis Research Unit, Aarhus University Hospital, Risskov, Aarhus, DK
129. University of Liverpool, Liverpool, GB
130. Mental Health Center Copenhagen, Copenhagen University Hospital, Copenhagen, DK
131. Human Genetics and Computational Biomedicine, Pfizer Global Research and Development, Groton, CT, US
132. Psychiatry, Harvard Medical School, Boston, MA, US
133. Psychiatry, University of Iowa, Iowa City, IA, US
134. Department of Psychiatry and Behavioral Sciences, Johns Hopkins University, Baltimore, MD, US
135. Department of Psychiatry and Psychotherapy, University Medical Center Göttingen, Goettingen, Niedersachsen, DE
136. Human Genetics Branch, NIMH Division of Intramural Research Programs, Bethesda, MD, US
137. Faculty of Medicine, University of Iceland, Reykjavik, IS
138. Child and Adolescent Psychiatry, Erasmus MC, Rotterdam, Zuid-Holland, NL
139. Psychiatry, Erasmus MC, Rotterdam, Zuid-Holland, NL
140. Psychiatry, Dalhousie University, Halifax, NS, CA
141. Division of Epidemiology, New York State Psychiatric Institute, New York, NY, US
142. Department of Clinical Medicine, University of Copenhagen, Copenhagen, DK
143. Department of Medical & Molecular Genetics, King's College London, London, GB
144. Psychiatry & Behavioral Sciences, Stanford University, Stanford, CA, US
145. NIHR BRC for Mental Health, King's College London, London, GB
146. Genetics, University of North Carolina at Chapel Hill, Chapel Hill, NC, US
147. Psychiatry, University of North Carolina at Chapel Hill, Chapel Hill, NC, US

Cohort description

Participants were sampled from the Generation Scotland: Scottish Family Health Study (GS:SFHS) – a family-based epidemiological cohort recruited at random from General Practitioners' practices throughout Scotland between 2006 and 2011 (Smith *et al.*, 2013, Smith *et al.*, 2006). During baseline assessment, participants aged 18-98 (N = 24,090, Mean = 47.64, SD = 15.41) provided a wealth of clinical, phenotypic and biological data, including personality measures such as Neuroticism and a structured interview for clinical MDD diagnosis. Blood and salivary DNA was also taken for 98% of the cohort for genome-wide genotyping (Smith et al, 2006; Smith et al, 2013). In September 2014, GS:SFHS participants were re-contacted and asked to take part in a follow-up assessment of mental health and resilience (Navrady *et al.*, 2017). A total of 9,618 participants aged 22-100 (Mean = 56.43, SD = 13.37) provided useable re-contact data including questionnaire measures of self-reported MDD and resilience. This study includes 4,166 unrelated individuals (Mean age = 56.01, SD = 12.31, n female = 2,634) with complete data of interest.

References

Smith B, Campbell H, Blackwood D, Connell J, Connor M, Deary I, et al. (2006): Generation Scotland: the Scottish Family Health Study; a new resource for researching genes and heritability. *BMC Med Genet*. 7:74.

Smith BH, Campbell A, Linksted P, Fitzpatrick B, Jackson C, Kerr SM, et al. (2013): Cohort Profile: Generation Scotland: Scottish Family Health Study (GS:SFHS). The study, its participants and their potential for genetic research on health and illness. *Int J Epidemiol*. 42:689-700.

Navrady L, Wolters M, MacIntyre D, Clarke T, Campbell A, Murray A, et al. (2017): Cohort Profile: Stratifying Resilience and Depression Longitudinally (STRADL): a questionnaire follow-up of Generation Scotland: Scottish Family Health Study (GS:SFHS). *Int J Epidemiol*.doi: https://doi.org/10.1093/ije/dyx1115.

Thresholds for MDD Polygenic Risk Scores

Supplementary Table 1. Results of generalized linear mixed models predicting odds ratios for clinical MDD status (SCID), *p* value, upper and lower 95% confidence intervals and the Akaike Information Criterion, from five PRS thresholds

PRS Threshold	Odds Ratio	Lower 95% CI	Upper 95% CI	*p* value	AIC
0.01	1.24	1.14	1.35	< 0.001	3600.90
0.05	1.25	1.15	1.37	< 0.001	3598.40
0.10	1.26	1.16	1.37	< 0.001	3597.50
0.50	1.20	1.11	1.31	< 0.001	3607.60
1.00	1.20	1.10	1.31	< 0.001	3608.20

Abbreviations: SCID, Structured Clinical Interview for DSM-IV Axis I Disorders, representing clinical MDD; MDD, Major Depressive Disorder; AIC, Akaike Information Criterion; PRS, Polygenic Risk Score

NB. Each model was adjusted for baseline age (t_1: when the SCID was administered), sex and four principal components which control for population stratification

Supplementary Table 2. Results of generalized linear mixed models predicting odds ratios of self-reported MDD status (CIDI-SF), *p* value, upper and lower 95% confidence intervals and the Akaike Information Criterion, from five PRS thresholds

PRS Threshold	Odds Ratio	Lower 95% CI	Upper 95% CI	*p* value	AIC
0.01	1.13	1.05	1.21	0.001	4642.80
0.05	1.14	1.06	1.22	< 0.001	4640.90
0.10	1.16	1.08	1.25	< 0.001	4636.00
0.50	1.18	1.10	1.27	< 0.001	4632.80
1.00	1.18	1.10	1.27	< 0.001	4633.20

Abbreviations: CIDI-SF, Composite International Diagnostic Interview – Short Form, representing self-reported MDD; MDD, Major Depressive Disorder; AIC, Akaike Information Criterion; PRS, Polygenic Risk Score

NB. Each model was adjusted for age at follow-up (t_2: when the CIDI-SF was completed), sex and four principal components which control for population stratification

Supplementary Table 3. Correlation Matrix and Descriptive Statistics

	BRS_1	BRS_2	BRS_3	BRS_4	BRS_5	BRS_6	Resilience	EPQ_1	EPQ_3	EPQ_5	EPQ_7	EPQ_9	EPQ_{11}	EPQ_{13}	EPQ_{15}	EPQ_{17}	EPQ_{19}	EPQ_{21}	EPQ_{23}	Neuroticism	Mean (SD)	N (%)
BRS_1	-																				3.70 (0.96)	
BRS_2	.47	-																			3.40 (1.04)	
BRS_3	.68	.45	-																		3.49 (1.00)	
BRS_4	.56	.63	.51	-																	3.52 (1.04)	
BRS_5	.62	.50	.63	.52	-																3.55 (1.01)	
BRS_6	.60	.61	.54	.69	.57	-															3.64 (1.04)	
Resilience	.81	.77	.79	.82	.80	.84	-														3.52 (0.82)	
EPQ_1	-.26 **	-.24 **	-.27 **	-.24 **	-.28 **	-.24 **	-.32 **	-														1513 (36)
EPQ_3	-.30 **	-.26 **	-.28 **	-.26 **	-.29 **	-.27 **	-.34 **	.63 *	-													1310 (31)
EPQ_5	-.15 **	-.14 **	-.14 **	-.13 **	-.15 **	-.14 **	-.18 **	.52 *	.36 *	-												1105 (27)
EPQ_7	-.22 **	-.20 **	-.23 **	-.23 **	-.23 **	-.22 **	-.27 **	.40 *	.42 *	.30 *	-											1847 (44)
EPQ_9	-.30 **	-.27 **	-.27 **	-.26 **	-.27 **	-.27 **	-.34 **	.70 *	.66 *	.47 *	.45 *	-										1276 (31)
EPQ_{11}	-.24 **	-.24 **	-.23 **	-.23 **	-.24 **	-.24 **	-.30 **	.43 *	.40 *	.36 *	.47 *	.40 *	-									990 (24)
EPQ_{13}	-.27 **	-.27 **	-.27 **	-.27 **	-.30 **	-.25 **	-.34 **	.50 *	.47 *	.39 *	.57 *	.49 *	.58 *	-								2382 (57)
EPQ_{15}	-.23 **	-.22 **	-.20 **	-.20 **	-.20 **	-.20 **	-.26 **	.52 *	.43 *	.50 *	.47 *	.44 *	.69 *	.66 *	-							653 (16)
EPQ_{17}	-.23 **	-.20 **	-.21 **	-.22 **	-.24 **	-.20 **	-.27 **	.40 *	.42 *	.28 *	.60 *	.43 *	.50 *	.62 *	.46 *	-						1796 (43)
EPQ_{19}	-.23 **	-.24 **	-.22 **	-.22 **	-.24 **	-.23 **	-.29 **	.47 *	.43 *	.38 *	.41 *	.46 *	.75 *	.61 *	.70 *	.43 *	-					848 (20)
EPQ_{21}	-.22 **	-.18 **	-.19 **	-.18 **	-.20 **	-.21 **	-.25 **	.57 *	.55 *	.36 *	.44 *	.67 *	.38 *	.42 *	.45 *	.40 *	.43 *	-				582 (14)
EPQ_{23}	-.24 **	-.20 **	-.24 **	-.23 **	-.23 **	-.21 **	-.28 **	.49 *	.50 *	.34 *	.51 *	.48 *	.48 *	.59 *	.49 *	.66 *	.48 *	.49 *	-			1107 (27)
Neuroticism	-.41	-.37	-.39	-.38	-.40	-.38	-.48	.64 **	.60 **	.49 **	.59 **	.64 **	.60 **	.64 **	.58 **	.61 **	.59 **	.51 **	.61 **	-	3.70 (3.17)	
Aget1	.04	.04	.04	.03	.04	.05	.05	-.15 **	-.13 **	-.14 **	-.04 **	-.14 **	-.06 **	-.04 **	-.04 **	-.06 **	-.12 **	-.02 **	-.05 **	-.14	50.28 (12.34)	
Aget2	.05	.04	.04	.03	.04	.05	.05	-.15 **	-.13 **	-.13 **	-.03 **	-.13 **	-.06 **	-.04 **	-.04 *	-.06 **	-.12 **	-.01 **	-.04 **	-.14	56.01 (12.31)	
Sex (F)	-.10 **	-.06 **	-.09 **	-.09 **	-.11 **	-.06 **	-.10 **	.10 *	.22 *	-.06 *	.37 *	.06 *	.16 *	.26 *	.15 *	.27 *	.04 *	.20 *	.26 *	.17 **		2634 (63)
SCID	-.27 **	-.23 **	-.24 **	-.23 **	-.28 **	-.24 **	-.31 **	.45 *	.51 *	.22 *	.33 *	.43 *	.37 *	.39 *	.38 *	.32 *	.41 *	.47 *	.42 *	.36 **		664 (16)
CIDI-SF	-.31 **	-.26 **	-.29 **	-.26 **	-.31 **	-.28 **	-.35 **	.35 *	.42 *	.16 *	.22 *	.37 *	.26 *	.26 *	.26 *	.24 *	.29 *	.39 *	.33 *	.29 **		1068 (26)

Abbreviations: Age_{t1}, Age at baseline; Age_{t2}, Age at re-contact; BRS, Individual items from the Brief Resilience Scale; Resilience, Total score from the Brief Resilience Scale; EPQ, Individual items from the Eysenck Personality Questionnaire Short-Form; Neuroticism, Total score from the Eysenck Personality Questionnaire Short-Form; SCID, Structured Clinical Interview for DSM-IV Axis I Disorders representing clinical MDD; CIDI-SF, Composite International Diagnostic Interview – Short Form representing self-reported MDD.

N.B. All p-values significant at $p \leq 0.01$.

EPQ items represent the number and percentage of 'Yes' responses. SCID and CIDI-SF represent the number and percentage of individuals meeting criteria for clinical and self-reported MDD, respectively.

All coefficients represent Pearson correlations except those denoted by * which represent tetrachoric correlations – resultant from both variables being binary, and those denoted by ** which represent point biserial correlations – resultant from binary and continuous variables

Descriptive Statistics for Clinical and Self-reported MDD

Clinical MDD

Based on Structured Clinical Interview for DSM-IV Axis I Disorders (SCID; First *et al.*, 1997) at baseline assessment (2006-2011), clinically diagnosed MDD cases were predominately female (73%) and younger than non-MDD cases (61% female, M = 49.13, SD = 11.39 and M = 50.49, SD = 12.50, respectively); ($t(990.30) = 2.78$, $p<0.001$, Cohen's d = .11). Clinical MDD cases were found to have significantly higher neuroticism scores (M = 6.31, SD = 3.28) than did non-MDD cases (M = 3.20, SD = 2.90); ($t(869.91) = 22.75$, $p < 0.001$, Cohen's d = 1.00). Clinically diagnosed MDD cases were found to score significantly lower in resilience (M = 2.94, SD = 0.85) in comparison to non-MDD cases (M = 3.63, SD = 0.76); ($t(879.19) = 19.48$, $p < 0.001$, Cohen's d = .08).

Self-reported MDD

Using the Composite International Diagnostic Interview – Short Form (CIDI-SF; Kessler *et al.*, 1998) at re-contact (2014-2017), a larger proportion of females met criteria for self-reported depression (75%) in comparison to non-MDD cases (59%). Self-reported MDD cases were younger (M = 54.40, SD = 12.28) in comparison to non-MDD cases (M = 56.56, SD = 12.27) at re-contact; ($t(1852.70) = 4.96$, $p < 0.001$, Cohen's d = .18). Individuals self-reporting MDD scored higher in neuroticism than did non-MDD cases (M = 5.26, SD = 3.45 and M = 3.16, SD = 2.88, respectively); ($t(1608) = 17.80$, $p < 0.001$, Cohen's d = .66). Significant group differences were found between self-reported MDD cases (M = 3.03, SD = 0.86) and non-MDD cases (M = 3.69, SD = 0.73) in resilience; ($t(1617.60) = 22.33$, $p < 0.001$, Cohen's d = .83), whereby self-reported MDD cases scored lower on psychological resilience.

Overlap between clinical and self-reported MDD

The two measures of MDD were taken at two separate time-points using two different methods, approximately six years apart. Below is a table detailing the overlap between these two measures in our sample of 4,166 individuals.

Supplementary Table 4

Table demonstrating the overlap of individuals meeting criteria for clinical and self-reported MDD in the current sample (n = 4,166)

		Self-reported MDD (CIDI-SF)	
		Met criteria	Did not meet criteria
Clinical MDD (SCID)	Met criteria	411	253
	Did not meet criteria	6157	2,845

Abbreviations: SCID, Structured Clinical Interview for DSM-IV Axis I Disorders, representing clinical MDD; CIDI-SF, Composite International Diagnostic Interview – Short Form, representing self-reported MDD; MDD, Major Depressive Disorder

References

First, M., Spitzer, R., Gibbon, M. & Williams, J. (1997). Structured Clinical Interview for DSM-IV Axis I Disorders, Clinician Version (SCID-CV). American Psychiatric Publishing, Inc.: Washington, DC, USA.

Kessler, R., Andrews, G., Mroczek, D., Ustun, B. & Wittcjen, H.-U. (1998). The World Health Organisation Composite International Diagnostic Interview short-form (CIDI-SF). pp. 171-185. International Journal of Methods in Psychiatric Research.

Moderation models for clinical MDD

Supplementary Table 5. Results of a generalised linear model predicting odds ratios for clinical MDD status, *p* value, upper and lower 95% confidence intervals and the Akaike Information Criterion

MDD Outcome	Variables	Odds ratio	Lower 95% CIs	Upper 95% CIs	*p* value	AIC
SCID	Age_{t1}	0.99	0.99	1.00	0.031	3607.60
	Sex (F)	1.71	1.42	2.07	$1.53x10^{-8}$	
	PRS	1.20	1.11	1.31	$1.87x10^{-5}$	
	C1	2.92	0.00	$7.89x10^{24}$	0.969	
	C2	0.01	0.00	$8.63x10^{26}$	0.886	
	C3	0.00	0.00	1.05	0.127	
	C4	$1.83x10^{4}$	0.01	$2.07x10^{10}$	0.169	
SCID	Age_{t1}	0.99	0.99	1.00	0.026	3155.90
	Sex (F)	1.33	1.09	1.63	0.005	
	PRS	1.16	1.05	1.29	0.004	
	Neuroticism	2.49	2.28	2.72	$< 2.00x10^{-16}$	
	PRS * Neuroticism	0.92	0.84	1.00	0.062	
	C1	$1.60x10^{3}$	0.00	$2.18x10^{29}$	0.805	
	C2	0.00	0.00	$2.05x10^{26}$	0.431	
	C3	0.01	0.00	$8.08x10^{2}$	0.516	
	C4	134.63	0.00	$3.43x10^{8}$	0.062	
SCID	Age_{t1}	0.99	0.99	1.00	0.030	3251.90
	Sex (F)	1.53	1.26	1.86	$1.97x10^{-5}$	
	PRS	1.17	1.06	1.30	0.002	
	Resilience	0.44	0.40	0.48	$< 2.00x10^{-16}$	
	PRS * Resilience	1.06	0.97	1.16	0.211	
	C1	0.00	0.00	$1.58x10^{22}$	0.753	
	C2	0.00	0.00	$8.81x10^{22}$	0.629	
	C3	0.00	0.00	$2.42x10^{2}$	0.323	
	C4	$1.03x10^{48}$	0.04	$2.44x10^{11}$	0.124	

Abbreviations: SCID, Structured Clinical Interview for DSM-IV Axis I Disorders, representing clinical MDD; MDD, Major Depressive Disorder; AIC, Akaike Information Criterion; PRS, Polygenic Risk Score; Age_{t1}, Age at the time of baseline; C1-4, the principal components which control for population stratification
N.B. Neuroticism has been controlled for Age_{t1} and these residuals used within the model. Resilience has been controlled for Age_{t2} before entering the model. Threshold for PRS = 0.50

Moderation models for self-reported MDD

Supplementary Table 6. Results of a generalised linear model predicting odds ratios for self-reported MDD status, *p* value, upper and lower 95% confidence intervals and the Akaike Information Criterion

MDD Outcome	Variables	Odds ratio	Lower 95% CIs	Upper 95% CIs	*p* value	AIC
CIDI-SF	Age_{t2}	0.99	0.98	0.99	$1.88x10^{-5}$	4632.80
	Sex (F)	1.94	1.66	2.28	$< 2.00x10^{-16}$	
	PRS	1.18	1.10	1.27	$6.01x10^{-6}$	
	C1	$5.87x10^{18}$	0.01	$2.18x10^{40}$	0.082	
	C2	0.63	0.00	$3.17x10^{24}$	0.987	
	C3	0.00	0.00	1.85	0.068	
	C4	$5.41x10^{2}$	0.00	$7.40x10^{7}$	0.298	
CIDI-SF	Age_{t2}	0.99	0.98	0.99	$4.48x10^{-6}$	4366.40
	Sex (F)	1.66	1.41	1.95	$1.03x10^{-9}$	
	PRS	1.13	1.05	1.22	0.002	
	Neuroticism	1.81	1.68	1.95	$< 2.00x10^{-16}$	
	PRS * Neuroticism	0.97	0.90	1.04	0.416	
	C1	$1.81x10^{21}$	0.54	$4.30x10^{43}$	0.058	
	C2	0.07	0.00	$3.19x10^{24}$	0.931	
	C3	0.00	0.00	20.120	0.187	
	C4	19.49	0.00	$3.89x10^{6}$	0.634	
CIDI-SF	Age_{t2}	0.99	0.98	0.99	$4.15x10^{-6}$	4156.80
	Sex (F)	1.80	1.52	2.12	$4.38x10^{-12}$	
	PRS	1.14	1.06	1.24	0.001	
	Resilience	0.43	0.40	0.47	$< 2.00x10^{-16}$	
	PRS * Resilience	1.07	0.99	1.17	0.080	
	C1	$3.75x10^{17}$	0.00	$1.06x10^{41}$	0.134	
	C2	0.00	0.00	$4.61x10^{21}$	0.733	
	C3	0.00	0.00	16.60	0.168	
	C4	$1.28x10^{3}$	0.00	1.17	0.266	

Abbreviations: CIDI-SF, Composite International Diagnostic Interview – Short Form, representing self-reported MDD; MDD, Major Depressive Disorder; AIC, Akaike Information Criterion; PRS, Polygenic Risk Score; Age_{t2}, Age at the time of re-contact; C1-4, the principal components which control for population stratification

N.B. Neuroticism has been controlled for Age_{t1} and these residuals used within the model. Resilience has been controlled for Age_{t2}, before entering the model. Threshold for PRS = 0.50

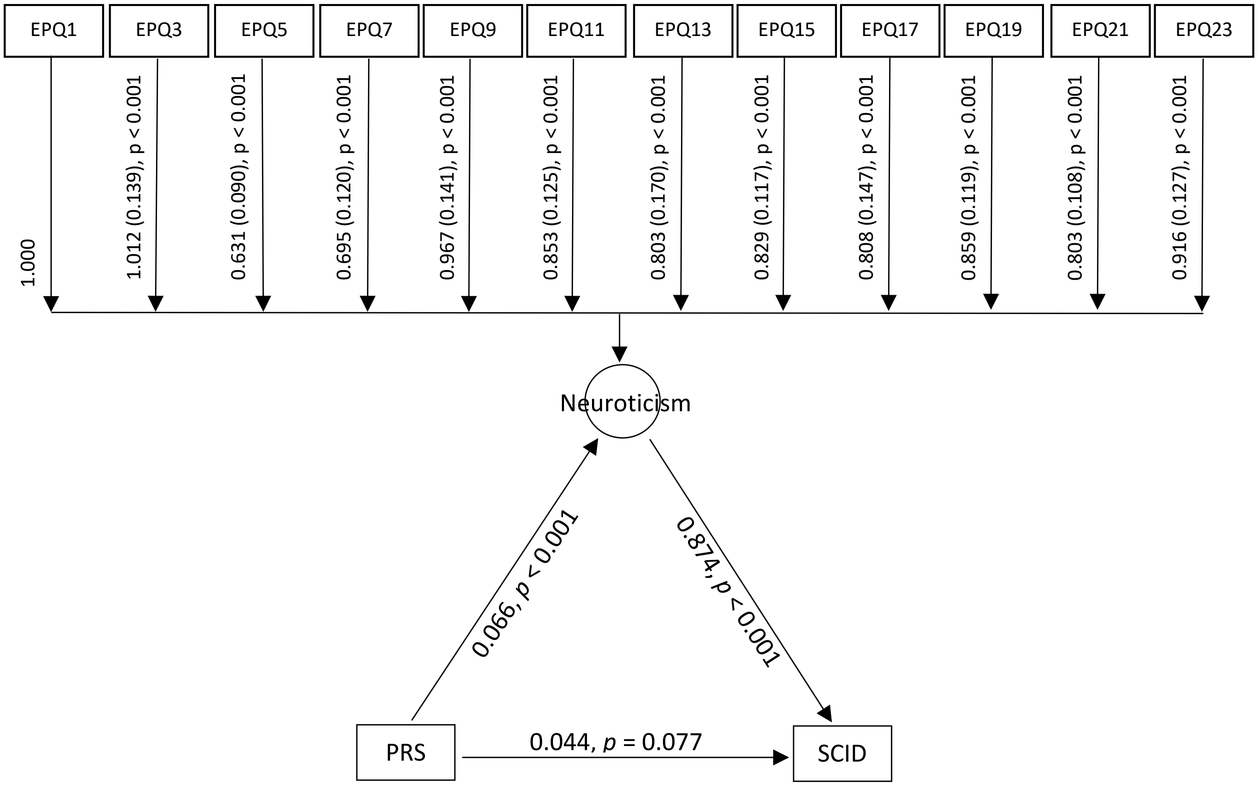

Supplementary Figure 1. Path diagram of **Model 1A**, which includes factor loadings onto the latent variable neuroticism, a direct path between PRS and clinical MDD status, and indirect path through neuroticism. Values are standarised path coefficients. All endogenous variables have been adjusted for population stratification, sex and the age at which the variable was measured.

Abbreviations: PRS, Polygenic Risk Score; SCID, Structured Clinical Interview for DSM-IV Axis I Disorders, representing clinical MDD; MDD, Major Depressive Disorder; EPQ; the Eysenck Personality Questionnaire Short Form-Revised

Supplementary Table 7. Results of all standarised path coefficients from **Model 1A** examining the mediation of Neuroticism through PRS to clinical MDD status

Model description		ß	S.E	*p* value
SCID ~	PRS	0.044	0.025	0.077
SCID ~	Neuroticism	0.874	0.114	< 0.001
SCID ~	Sex	0.191	0.054	< 0 .001
SCID ~	Age_{t1}	0.003	0.027	0.900
SCID ~	C1	0.004	0.024	0.856
SCID ~	C2	-0.004	0.025	0.868
SCID ~	C3	-0.020	0.025	0.429
SCID ~	C4	0.013	0.025	0.605
Neuroticism ~	PRS	0.066	0.010	< 0 .001
Neuroticism ~	Sex	0.116	0.019	< 0 .001
Neuroticism ~	Age_{t1}	-0.063	0.010	< 0 .001
Neuroticism ~	C1	-0.004	0.005	0.514
Neuroticism ~	C2	0.001	0.005	0.828
Neuroticism ~	C3	-0.020	0.006	0 .001
Neuroticism ~	C4	0.022	0.006	< 0 .001

Abbreviations: SCID, Structured Clinical Interview for DSM-IV Axis I Disorders, representing clinical MDD; PRS, Polygenic Risk Score; Age_{t1}, Age at baseline; C1-4, the principal components which control for population stratification

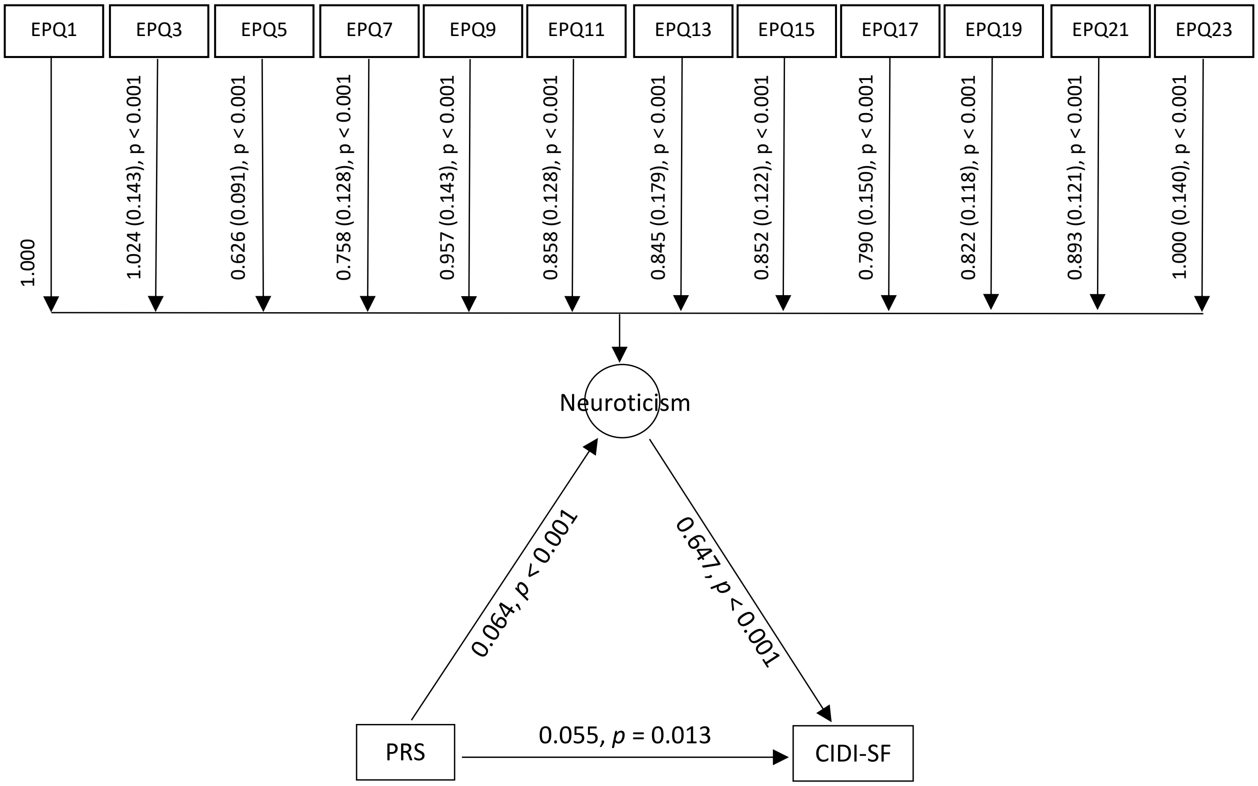

Supplementary Figure 2. Path diagram of **Model 2A**, which includes factor loadings onto the latent variable neuroticism, a direct path between PRS and self-reported MDD status, and indirect path through neuroticism. Values are standarised path coefficients. All endogenous variables have been adjusted for population stratification, sex and the age at which the variable was measured.

Abbreviations: PRS, Polygenic Risk Score; CIDI-SF, Composite International Diagnostic Interview – Short Form, representing self-reported MDD; MDD, Major Depressive Disorder; EPQ; the Eysenck Personality Questionnaire Short Form-Revised

Supplementary Table 8. Results of all standarised path coefficients from **Model 2A** examining the mediation of Neuroticism through PRS to self-reported MDD status

Model description		ß	S.E	*p* value
CIDI-SF ~	PRS	0.055	0.022	0.013
CIDI-SF ~	Neuroticism	0.647	0.087	< 0.001
CIDI-SF ~	Sex	0.312	0.047	< 0.001
CIDI-SF ~	Age_{t2}	-0.030	0.188	0.874
CIDI-SF ~	C1	0.045	0.024	0.065
CIDI-SF ~	C2	-0.002	0.022	0.931
CIDI-SF ~	C3	-0.028	0.022	0.203
CIDI-SF ~	C4	0.008	0.022	0.705
Neuroticism ~	PRS	0.064	0.010	< 0.001
Neuroticism ~	Sex	0.119	0.020	< 0.001
Neuroticism ~	Age_{t1}	-0.249	0.055	< 0.001
Neuroticism ~	C1	-0.003	0.005	0.540
Neuroticism ~	C2	0.002	0.005	0.719
Neuroticism ~	C3	-0.020	0.006	0.001
Neuroticism ~	C4	0.021	0.006	0.001

Abbreviations: CIDI-SF, Composite International Diagnostic Interview – Short Form, representing self-reported MDD; PRS, Polygenic Risk Score; Age_{t1}, Age at baseline; Age_{t2}, Age at re-contact; C1-4, the principal components which control for population stratification

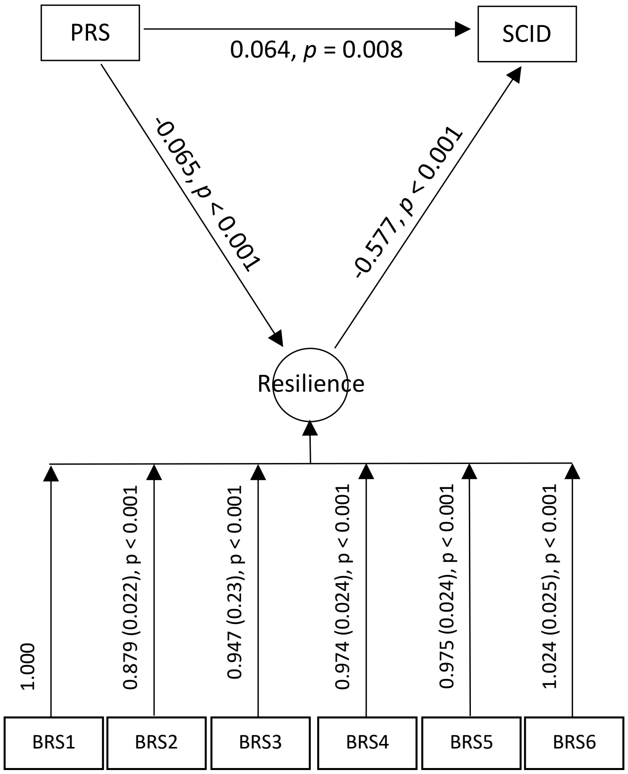

Supplementary Figure 3. Path diagram of **Model 1B**, which includes factor loadings onto the latent variable resilience, a direct path between PRS and clinical MDD status, and indirect path through resilience. Values are standarised path coefficients. All endogenous variables have been adjusted for population stratification, sex and the age at which the variable was measured.

Abbreviations: PRS, Polygenic Risk Score; SCID, Structured Clinical Interview for DSM-IV Axis I Disorders, representing clinical MDD; MDD, Major Depressive Disorder; BRS; the Brief Resilience Scale

Supplementary Table 9. Results of all standarised path coefficients from **Model 1B** examining the mediation of Resilience through PRS to clinical MDD status

Model description		ß	S.E	*p* value
SCID ~	PRS	0.064	0.024	0.008
SCID ~	Resilience	-0.577	0.018	< 0.001
SCID ~	Sex	0.197	0.052	< 0.001
SCID ~	Age_{t1}	-0.452	0.201	0.024
SCID ~	C1	-0.008	0.024	0.741
SCID ~	C2	-0.010	0.025	0.697
SCID ~	C3	-0.028	0.025	0.271
SCID ~	C4	0.030	0.025	0.228
Resilience ~	PRS	-0.577	0.018	< 0.001
Resilience ~	Sex	-0.165	0.014	< 0.001
Resilience ~	Age_{t2}	0.010	0.056	0.860
Resilience ~	C1	-0.015	0.007	0.021
Resilience ~	C2	-0.014	0.007	0.040
Resilience ~	C3	0.018	0.007	0.006
Resilience ~	C4	-0.004	0.007	0.531

Abbreviations: SCID, Structured Clinical Interview for DSM-IV Axis I Disorders, representing clinical MDD; PRS, Polygenic Risk Score; Age_{t1}, Age at baseline; Age_{t2}, Age at re-contact; C1-4, the principal components which control for population stratification

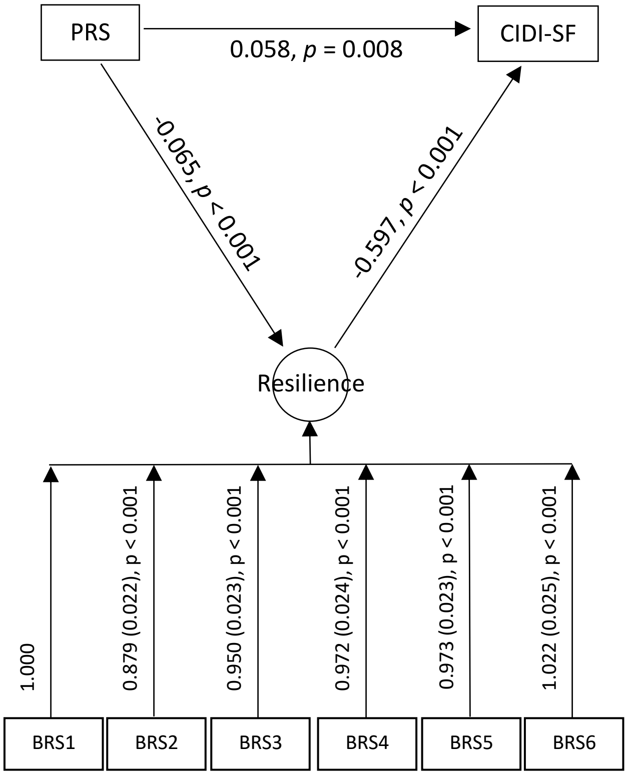

Supplementary Figure 4. Path diagram of **Model 2B**, which includes factor loadings onto the latent variable resilience, a direct path between PRS and self-reported MDD status, and indirect path through resilience. Values are standarised path coefficients. All endogenous variables have been adjusted for population stratification, sex and the age at which the variable was measured.

Abbreviations: PRS, Polygenic Risk Score; CIDI-SF, Composite International Diagnostic Interview – Short Form, representing self-reported MDD; MDD, Major Depressive Disorder; BRS; the Brief Resilience Scale

Supplementary Table 10. Results of all standarised path coefficients from **Model 2B** examining the mediation of Resilience through PRS to self-reported MDD status

Model description		ß	S.E	*p* value
CIDI-SF ~	PRS	0.058	0.022	0.008
CIDI-SF ~	Resilience	-0.597	0.018	< 0.001
CIDI-SF ~	Sex	0.290	0.047	< 0.001
CIDI-SF ~	Age_{t2}	-0.070	0.022	0.001
CIDI-SF ~	C1	0.033	0.024	0.171
CIDI-SF ~	C2	-0.009	0.022	0.683
CIDI-SF ~	C3	-0.030	0.022	0.177
CIDI-SF ~	C4	0.019	0.022	0.383
Resilience ~	PRS	-0.065	0.007	< 0.001
Resilience ~	Sex	-0.165	0.014	< 0.001
Resilience ~	Age_{t2}	0.037	0.006	< 0.001
Resilience ~	C1	-0.015	0.007	0.021
Resilience ~	C2	-0.014	0.007	0.040
Resilience ~	C3	0.018	0.007	0.006
Resilience ~	C4	-0.004	0.007	0.531

Abbreviations: CIDI-SF, Composite International Diagnostic Interview – Short Form, representing self-reported MDD; PRS, Polygenic Risk Score; Age_{t2}, Age at re-contact; C1-4, the principal components which control for population stratification

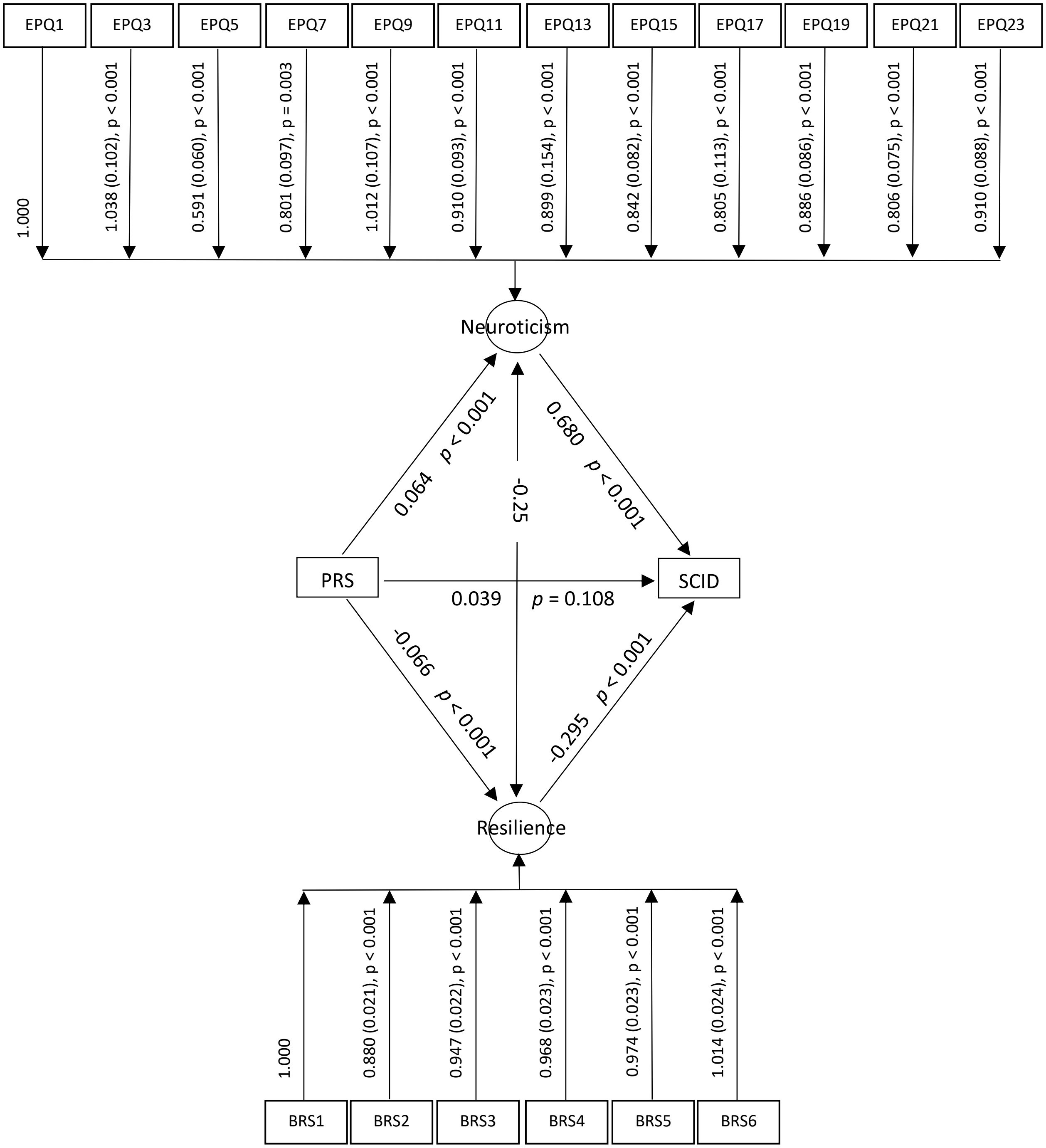

Supplementary Figure 5. Path diagram of **Model 1C**, which includes factor loadings onto the latent variables neuroticism and resilience, a direct bath between PRS and clinical MDD status, an indirect path through neuroticism and an indirect path through resilience. Values are standarised path coefficients. All endogenous variables have been adjusted for population stratification, sex and the age at which the variable was measured.

Abbreviations: PRS, Polygenic Risk Score; SCID, Structured Clinical Interview for DSM-IV Axis I Disorders, representing clinical MDD; MDD, Major Depressive Disorder; EPQ, the Eysenck Personality Questionnaire Short Form-Revised; BRS, the Brief Resilience Scale

Supplementary Table 11. Results of all standarised path coefficients from **Model 1C** examining the separate mediation of Neuroticism and Resilience through PRS to clinical MDD status

Model description		ß	S.E	*p* value
SCID ~	PRS	0.039	0.024	0.108
SCID ~	Neuroticism	0.680	0.105	< 0.001
SCID ~	Resilience	-0.295	0.044	< 0.001
SCID ~	Sex	0.164	0.053	0.002
SCID ~	Age_{t1}	-0.283	0.205	0.166
SCID ~	C1	-0.001	0.024	0.962
SCID ~	C2	-0.007	0.025	0.784
SCID ~	C3	-0.019	0.025	0.440
SCID ~	C4	0.017	0.025	0.499
Neuroticism ~	PRS	0.064	0.008	< 0.001
Neuroticism ~	Sex	0.116	0.017	< 0.001
Neuroticism ~	Age_{t1}	-0.249	0.052	< 0.001
Neuroticism ~	C1	-0.003	0.005	0.511
Neuroticism ~	C2	0.001	0.005	0.782
Neuroticism ~	C3	-0.020	0.006	< 0.001
Neuroticism ~	C4	0.021	0.006	< 0.001
Resilience ~	PRS	-0.066	0.007	< 0.001
Resilience ~	Sex	-0.165	0.014	< 0.001
Resilience ~	Age_{t2}	0.019	0.057	0.733
Resilience ~	C1	-0.016	0.007	0.021
Resilience ~	C2	-0.014	0.007	0.040
Resilience ~	C3	0.018	0.007	0.006
Resilience ~	C4	-0.004	0.007	0.530

Abbreviations: SCID, Structured Clinical Interview for DSM-IV Axis I Disorders, representing clinical MDD; PRS, Polygenic Risk Score; $Age_{t1,}$ Age at baseline; Age_{t2}, Age at re-contact; C1-4, the principal components which control for population stratification

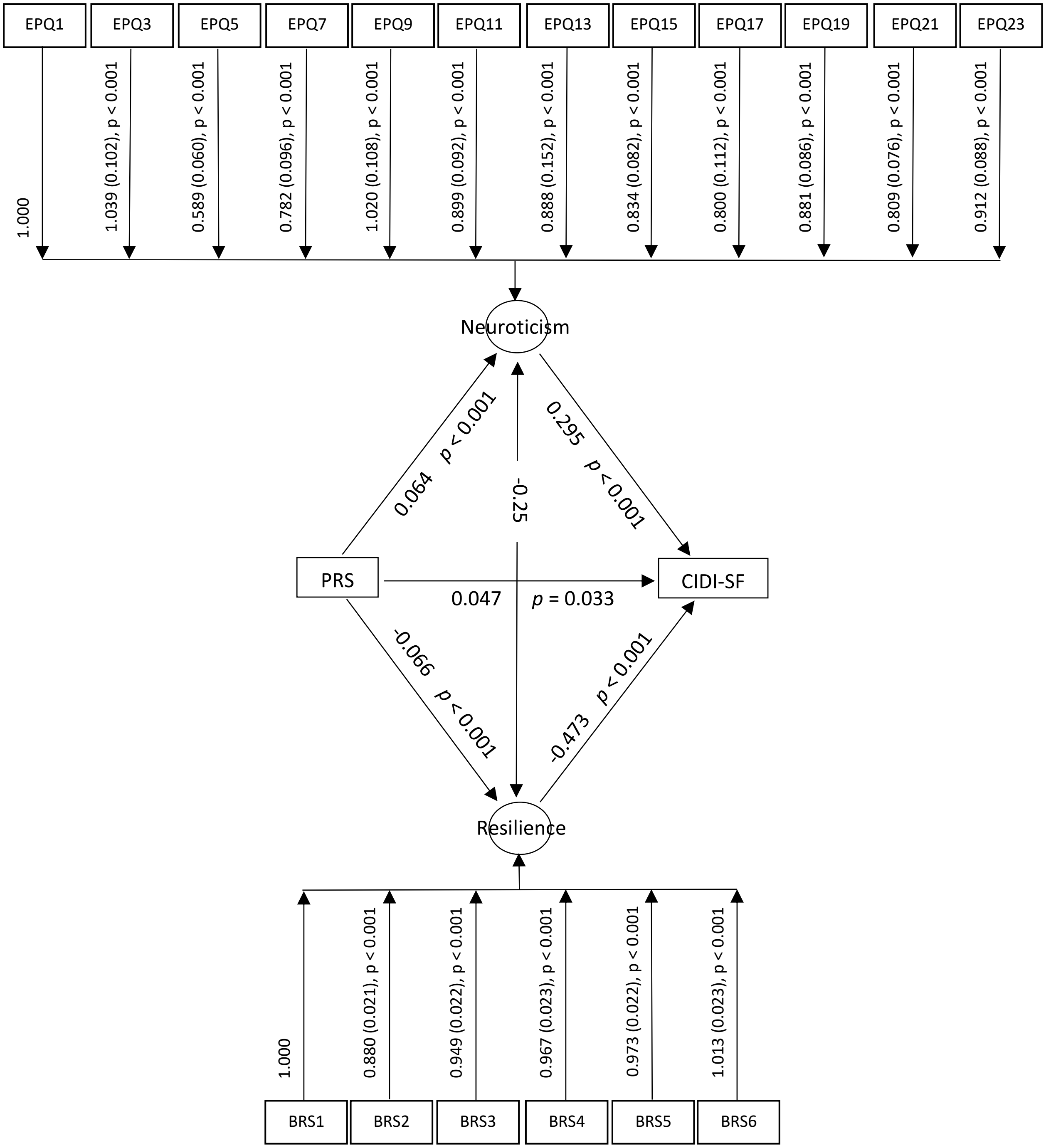

Supplementary Figure 6. Path diagram of **Model 2C**, which includes factor loadings onto the latent variables neuroticism and resilience, a direct bath between PRS and self-reported MDD status, an indirect path through neuroticism and an indirect path through resilience. Values are standarised path coefficients. All endogenous variables have been adjusted for population stratification, sex and the age at which the variable was measured.

Abbreviations: PRS, Polygenic Risk Score; CIDI-SF, Composite International Diagnostic Interview – Short Form, representing self-reported MDD; MDD, Major Depressive Disorder; EPQ, the Eysenck Personality Questionnaire Short Form-Revised; BRS, the Brief Resilience Scale

Supplementary Table 12. Results of all standarised path coefficients from **Model 2C** examining the separate mediation of Neuroticism and Resilience through PRS to self-reported MDD status

Model description		ß	S.E	*p* value
CIDI-SF ~	PRS	0.047	0.022	0.033
CIDI-SF ~	Neuroticism	0.295	0.070	< 0.001
CIDI-SF ~	Resilience	-0.473	0.035	< 0.001
CIDI-SF ~	Sex	0.276	0.047	< 0.001
CIDI-SF ~	Age_{t2}	-0.016	0.190	0.933
CIDI-SF ~	C1	0.036	0.024	0.136
CIDI-SF ~	C2	-0.008	0.022	0.730
CIDI-SF ~	C3	-0.027	0.022	0.231
CIDI-SF ~	C4	0.013	0.022	0.537
Neuroticism ~	PRS	0.064	0.008	< 0.001
Neuroticism ~	Sex	0.116	0.017	< 0.001
Neuroticism ~	Age_{t1}	-0.249	0.052	< 0.001
Neuroticism ~	C1	-0.003	0.005	0.513
Neuroticism ~	C2	0.001	0.005	0.783
Neuroticism ~	C3	-0.020	0.006	< 0.001
Neuroticism ~	C4	0.021	0.006	< 0.001
Resilience ~	PRS	-0.066	0.007	< 0.001
Resilience ~	Sex	-0.166	0.014	< 0.001
Resilience ~	Age_{t2}	0.029	0.057	0.608
Resilience ~	C1	-0.016	0.007	0.021
Resilience ~	C2	-0.014	0.007	0.040
Resilience ~	C3	0.018	0.007	0.006
Resilience ~	C4	-0.004	0.007	0.530

Abbreviations: Composite International Diagnostic Interview – Short Form, representing self-reported MDD; PRS, Polygenic Risk Score; $Age_{t1,}$ Age at baseline; Age_{t2}, Age at re-contact; C1-4, the principal components which control for population stratification

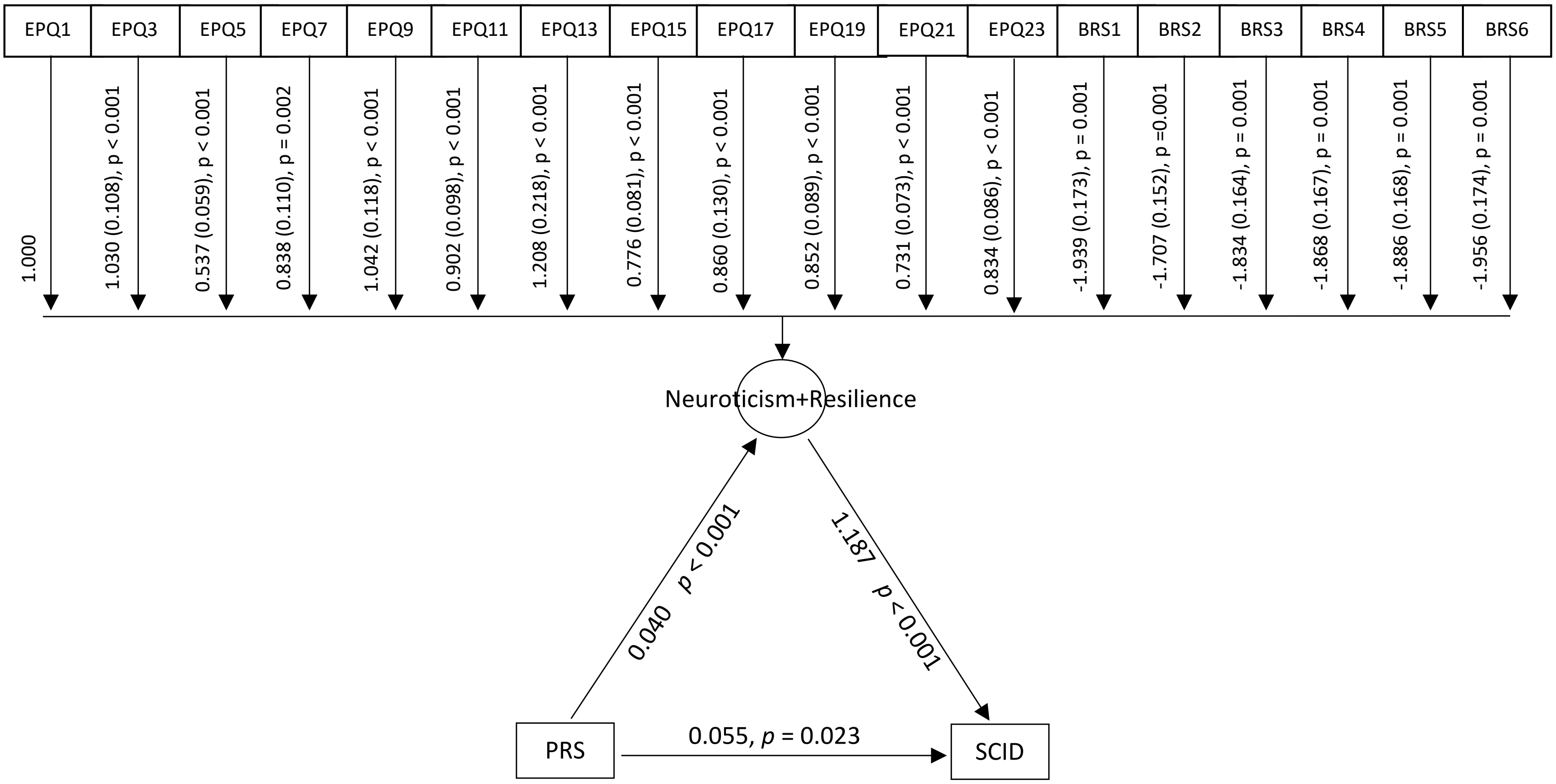

Supplementary Figure 7. Path diagram of **Model 1D**, which includes factor loadings onto the latent variable Neuroticism+Resilience, a direct path between PRS and clinical MDD status, and an indirect path through the latent variable (Neuroticism+Resilience). Values are standarised path coefficients. All endogenous variables have been adjusted for population stratification, sex and the age at which the variable was measured.

Abbreviations: PRS, Polygenic Risk Score; SCID, Structured Clinical Interview for DSM-IV Axis I Disorders, representing clinical MDD; MDD, Major Depressive Disorder; Neuroticism+Resilience, a latent factor comprised of the individual items from the Eysenck Personality Questionnaire Short Form-Revised and the individual items from the Brief Resilience Scale.

Supplementary Table 13. Results of all standarised path coefficients from **Model 1D** examining the mediation of a latent variable Neuroticism+Resilience through PRS to clinical MDD status

Model description		ß	S.E	*p* value
SCID ~	PRS	0.055	0.024	0.023
SCID ~	Neuroticism+Resilience	1.187	0.108	< 0.001
SCID ~	Sex	0.184	0.052	< 0.001
SCID ~	Age_{t1}	-0.371	0.203	0.067
SCID ~	C1	-0.004	0.024	0.856
SCID ~	C2	-0.008	0.025	0.748
SCID ~	C3	-0.024	0.025	0.341
SCID ~	C4	0.023	0.024	0.351
Neuroticism+Resilience ~	PRS	0.040	0.005	< 0.001
Neuroticism+Resilience ~	Sex	0.091	0.010	< 0.001
Neuroticism+Resilience ~	Age_{t1}	-0.068	0.026	0.008
Neuroticism+Resilience ~	Age_{t2}	0.042	0.025	0.093
Neuroticism+Resilience ~	C1	0.005	0.003	0.120
Neuroticism+Resilience ~	C2	0.005	0.003	0.073
Neuroticism+Resilience ~	C3	-0.012	0.003	< 0.001
Neuroticism+Resilience ~	C4	0.008	0.003	0.009

Abbreviations: Structured Clinical Interview for DSM-IV Axis I Disorders, representing clinical MDD ; PRS, Polygenic Risk Score; Age_{t1}, Age at baseline; Age_{t2}, Age at re-contact; C1-4, the principal components which control for population stratification; Neuroticism+Resilience, a latent factor comprised of the individual items from the Eysenck Personality Questionnaire Short Form-Revised and the individual items from the Brief Resilience Scale.

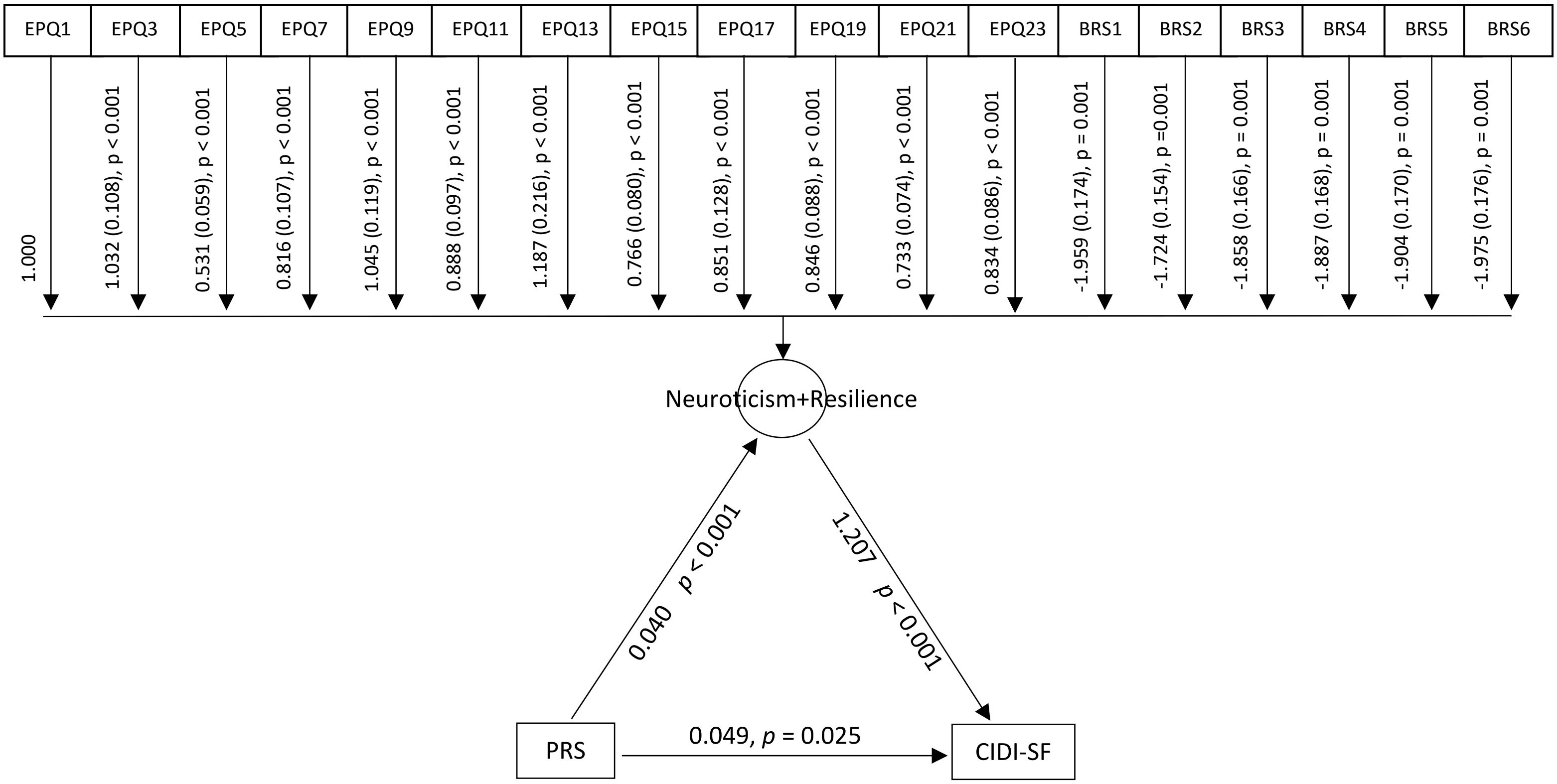

Supplementary Figure 12. Path diagram of **Model 2D**, which includes factor loadings onto the latent variable Neuroticism+Resilience, a direct path between PRS and self-reported MDD status, and an indirect path through the latent variable (Neuroticism+Resilience). Values are standarised path coefficients. All endogenous variables have been adjusted for population stratification, sex and the age at which the variable was measured.

Abbreviations: PRS, Polygenic Risk Score; Composite International Diagnostic Interview – Short Form, representing self-reported MDD; MDD, Major Depressive Disorder; Neuroticism+Resilience, a latent factor comprised of the individual items from the Eysenck Personality Questionnaire Short Form-Revised and the individual items from the Brief Resilience Scale.

Supplementary Table 14. Results of all standarised path coefficients from **Model 2D** examining the mediation of a latent variable Neuroticism+Resilience through PRS to self-reported MDD status

Model description		ß	S.E	*p* value
CIDI-SF ~	PRS	0.049	0.022	0.025
CIDI-SF ~	Neuroticism+Resilience	1.207	0.109	< 0.001
CIDI-SF ~	Sex	0.280	0.047	< 0.001
CIDI-SF ~	Age_{t2}	-0.071	0.190	0.708
CIDI-SF ~	C1	0.037	0.024	0.126
CIDI-SF ~	C2	-0.007	0.022	0.752
CIDI-SF ~	C3	-0.027	0.022	0.230
CIDI-SF ~	C4	0.012	0.022	0.569
Neuroticism+Resilience ~	PRS	0.040	0.005	< 0.001
Neuroticism+Resilience ~	Sex	0.090	0.010	< 0.001
Neuroticism+Resilience ~	Age_{t1}	-0.067	0.025	0.009
Neuroticism+Resilience ~	Age_{t2}	0.034	0.025	0.167
Neuroticism+Resilience ~	C1	0.005	0.003	0.116
Neuroticism+Resilience ~	C2	0.005	0.003	0.071
Neuroticism+Resilience ~	C3	-0.012	0.003	< 0.001
Neuroticism+Resilience ~	C4	0.008	0.003	0.010

Abbreviations: Composite International Diagnostic Interview – Short Form, representing self-reported MDD; PRS, Polygenic Risk Score; Age_{t1}, Age at baseline; Age_{t2}, Age at re-contact; C1-4, the principal components which control for population stratification; Neuroticism+Resilience, a latent factor comprised of the individual items from the Eysenck Personality Questionnaire Short Form-Revised and the individual items from the Brief Resilience Scale
